# 

**Published:** 1896-12

**Authors:** 


### DIRECTORY OF DENTAL SOCIETIES.

*American Dental Association*—Meets first Tuesday in August, 1896. At Saratoga, N.Y. Pres., J. Y. Crawford, Nashville, Tenn.; Rec. Sec., Geo. H. Cushing, Chicago.

*Southern Dental Association*—Next meeting will be held at Nashville, Tenn., November, 1896. Pres., H. E. Beach, Clarksville, Tenn. Sec., S. W. Foster, Decatur, Ala.

### STATE DENTAL SOCIETIES.

*Alabama Dental Association*—Meets annually. Next meeting at Selma, on the second Tuesday of April, 1896. Pres. A. A. Pearson, Montgomery; Sec. S. W. Foster, Decatur.

*California State Dental Association*—Next meeting will be held in San Francisco, second Tuesday in June, 1895. Pres., L. A. Teague, San Francisco; Rec. Sec., W. Z. King, San Francisco; Cor. Sec., C. E. Post, San Francisco.

*Colorado State Dental Society*—Meets 1st Tuesday in June, 1894, at Glenwood Springs. Pres. P. T. Smith, Denver; Sec., Sarah May Townsend, Denver.

*Connecticut State Dental Society*—Next meeting will be held in Hartford, third Tuesday in May, 1895. Pres. Chas. P. Graham, Middletown; Rec. Sec. Geo. L. Parmele, Hartford.

*Dental Society of the State of Maryland and District of Columbia*—Meets annually. Next meeting at Washington City, on the second Tuesday in October, 1893. Pres. B. F. Coy, of Baltimore: Sec. M. W. Foster, Baltimore.

*Florida State Dental Association*—Meets annually. Next meeting at Tampa, 1st Tuesday in May, 1895. Pres, W. A. McQuaig, Lake City; Cor. Sec. S. W. Allen, Tampa.

*Georgia State Dental Society*—Meets at Indian Spring, in 1895. Exact time to be decided later; Pres. W. W. Hill, Washington; Rec. Sec. S. H. McKee, Americus; Cor. Sec. O. H. McDonald, Atlanta.

*Illinois State Dental Society*—The next meeting will be held at Springfield, Ill., May 12 to 15, 1896. Pres. W. A. Stevens, Chicago., Sec. Louis Ottofy, Chicago.

*Indiana State Dental Society*—Will meet at Detroit, Mich., on the third Tuesday of June, 1895. Pres. D. S. Harrold, Elwood; Secretary, G. E. Hunt, Indianapolis.

*Iowa State Dental Society*—Meets annually. Next meeting at Iowa City, May, 7th to 10th, inclusive, 1895. Pres. J. S. Kulp, Muscatine; Rec. Sec. F. T. Breene, Iowa City.

*Kentucky State Dental Association*—Meets annually. Next meeting in Louisville, Ky., 3rd Tuesday in June, 1896. Pres., J. H. Letcher, Danville; Sec., J. H. Baldwin, Louisville.

*Kansas State Dental Association*—Pres. C. B. Reed, Topeka; Sec., W. W. West, Topeka. Next meeting will be held at Topeka, second Monday in May, 1895.

*Maine Dental Society*—Will meet on the third Tuesday in July, 1895, in Bangor. Pres. T E. Tibbetts, Rockland; Sec. F. A. Knowlton, Fairfield.

*Maryland State Dental Association*—Next annual meeting will be held (place not definitely known yet,) in April, 1895. Pres., Chas. C. Harris, Baltimore; Sec, W. W. Dunbracco, Baltimore.

*Massachusetts Dental Society*—Meets annually in Boston, in June. Pres. Josiah W. Ball, Boston; Rec. Sec., E. O. Kinsman, Cambridge.

*Michigan State Dental Association*—Meets annually. Next meeting at Detroit, on the 3rd., Tuesday of June, 1896. Pres., J. Ward House, Grand Rapids; Sec. ———. Treas. Geo. H. Mosher, Jackson.

*Mississippi Dental Association*—Next meeting date to be fixed, 1895, at Jackson, Miss. Pres. T. C. West, Natchez; Sec. L. N. Jeffries, Natchez.

*Minnesota State Society*—Pres. E. B. Weeks, Leitchfield; Sec., H. L. Cruttenden, St. Paul. Will meet in Winona, in September, 1896.

*Missouri Dental Association*—Meets at Bertle Springs, Mo., second Tuesday in July, 1895. Pres., J. T. Fry, Moberly; Sec., W. W. Carter, Sedalia.

*Nebraska State Dental Society.*—Next annual meeting will be held at Norfolk, third Tuesday in May, 1895. Pres., H. W. Shriver, Omaha; Cor. Sec., O. M. Huestis, Nebraska City.

*New Jersey State Dental Society*—Meets annually. The next meeting will be held August, 1896. Pres. R. M. Sanger, E. Orange; Sec. C. A. Meeker, Newark.

*New Hampshire Dental Society*—Meets Annually. Next meeting will be held in Concord, N. H.; time to be decided later. Pres. W. R. Blackstone, Manchester; Sec. Burton C. Russell, Keene.

*North Carolina State Dental Society*—Meets in Salisbury, on the second Tuesday in May, 1895. Pres. H. H. Harper, Kinston; Sec., J. E. Wyche, Greensboro.

*North Dakota State Dental Society*—The next meeting will be held at Fargo, May, 1895. Pres. E. M. Pierce, Hillsboro; Sec. C. L. Rose, Fargo.

*Ohio State Dental Society*—Meets in Columbus, first Tuesday in December, 1896. Pres. Hervey Barnes, Cleveland; Sec. L. P. Bethel, Kent.

*Pennsylvania State Dental Society*—Will hold its next annual meeting at Eagle's Mere, Pennsylvania, July 9th, 1895. Pres., Dr. J. A. Libbey, Pittsburgh; Cor. Sec., H. E. Roberts, Philadelphia.

*Rhode Island Dental Association*—Pres., C. M. Noble, Providence; Sec., H. W. Gillett, Newport, Meets quarterly, second Tuesday in July, October, January and April. Annual Meeting second Tuesday in July, at Newport.

*South Carolina State Dental Association*—Meets annually. Pres. J. C. Orland, Spartanburg; Cor. Sec. C. B. Colson, Charleston. Next meeting at Spartanburg, October, 1895.

*Tennessee Dental Association*—Meets annually. Next meeting at Knoxville, July 9, 1895. Pres. W. H. Richards, Knoxville; Rec Sec. W. P. Houston, Lawrenceburg; Cor. Sec. W. J. Morrison, Nashville.

*The Dental Society of the State of New York*—Meets annually on the second Wednesday in May. Next session at Albany, 8th and 9th of May, 1895. Pres. F. T. Van Woert, Brooklyn; Sec. C. S. Butler, Buffalo; Cor. Sec. R. Ottolengui, New York.

*Texas State Dental Association*—Meets in San Antonia, third Tuesday in May, 1894. Pres. A. A. Beville, Waco; Sec. Chas. B. Lewis, Ennis

*Vermont State Dental Society*—Meets annually. Next meeting will be held at Brandon, Vt., third Wednesday in March, 1895. Pres. W. H. Wright, Brandon; Sec. Thos. Mound, Rutland.

*Virginia State Dental Association*—Will meet in 1895. Exact time and place to be decided later. Pres. H. W. Campbell, Suffolk; Sec. J. Hall Moore, Richmond.

*Washington State Dental Society*—Meets Annually. Next meeting will be held at Seattle, in the middle of May, 1895. Pres. B. S. Scott, Ellenburg; Sec., R. B. Gentle, Olympia.

*Wisconsin State Dental Society*—Meets annually. Next meeting will be held at Madison, Wis., the third Tuesday in July, 1895. Pres., Chas. C. Chittenden, Madison; Sec., C. A. Southwell, Milwaukee.

*West Virginia State Society.*—The annual meeting will be held at Parkersburg, on the first Wednesday of October, 1894. Pres. J. N. Mahan, Charleston; Sec. Geo. I. Keener, Morgantown.

### LOCAL SOCIETIES.

*American Academy of Dental Science*—Meets annually in Boston, Mass., on the first Wednesday in October. Pres. J. H. Batchelder, Rec. Sec. William Y. Allen.

*Atktnsonian Dental Society of Chicogo*—Meets second Tuesday each month. Pres. J. G. Reed; Sec. C. E. Meehoff.

*Buffalo Dental Association*—Meets last Monday evening of each month. Annual meeting in June. Pres. F. H. Boswell; Sec. J. A. Stackhouse.

*Brooklyn Dental Society and Second District Society United*—Meets quarterly—Pres. T. H. Holly; Rec. Sec. A. N. Russell, Brooklyn.

*Central Dental Association of Northern New Jersey*—Meets bi-monthly; and annually. The next annual meeting will be held about the middle of February, 1895. Pres. Thomas Moore, Paterson; Sec. Wm. L. Fish, Newark.

*Chicago Dental Society*—Meets monthly. Pres. W. V. B. Ames; Sec. A. H. Peck;

*Chicago Dental Club*—Meets on the fourth Monday of each month. Pres. I. D. Sperling; Sec. E. L. Clifford.

*Cleveland Dental Society.*—Meets the first Monday of each month. Pres. J. R. Bell; Sec. J. F. Stephan.

*Connecticut Valley Dental Society*—Meets annually. Next meeting in Oct. 1895, time and place to be decided later. Pres. C. C. Barker, Meriden, Conn.; Sec. Geo. A. Maxfield, Holyoke, Mass.

*Detroit Dental Society*—Meets monthly, Pres. Geo. L. Field, Sec. A. W. Diack.

*Dental Society of Southwestern Michigan*—Meets at St. Joseph, October 8th and 9th, 1895. Pres. S. M. White; Sec. E. I. Backers.

*Eighth District Dental Society, N. Y.*—Next annual meeting April 24th, 1894, in Buffalo. Pres. F. J. Burkhart, Batavia; Sec., W. V. Grove, Buffalo.

*East Tennessee Dental Association*—Meets annually. Next meeting will be held at Harriman, second Tuesday in June 1895. Pres. F. A. Shotwell, Rogersville; Sec. and Treas. Geo. C. Brause, Harriman.

*Eastern Illinois Dental Society*—Meets annually.

*Fifth District Dental Society of N. Y.*—Meets semi-annually. Next annual meeting will be held in Utica, 2d Tuesday in April, 1895. Pres. Frank Jones, Syracuse; Cor. Sec. A. R. Cooke, Syracuse.

*First District Dental Society of the State of New York*—Meets annually. The next meeting will be held on the second Tuesday of April 1895. Pres., Victor Hugo Jackson, New York City; Sec., Benj. C. Nash, New York City.

*First District Dental Society of Illinois*—Next meeting will be held in Canton, second Tuesday in September, 1895. Pres. W. A. Johnston, Peoria; Sec. W. O. Butter, La Harpe.

*Hayden Dental Society of Chicago*—Meets third Monday each month. Pres. T. E. Powell; Sec. A. J. Oakey.

*Knoxville City Dental Society*—Meets monthly. President, J. T. Cazier; Sec. A. J Cottrell.

*Minnesota State Dental Society*—Annual Meeting will be held in St. Paul, 2nd Wednesday in Sept., 1895. Pres. C. H. Robinsin, Wabasha; Sec. W. L. Cruttenden, Northfield.

*Mississippi Valley Dental Society*—Meets annually at Cincinnati. Next meeting March, 1896. Pres., W. V. B. Ames, Chicago; Rec. Sec. H. T. Smith, Cincinnati; Cor. Sec. H. C. Matlack, Cincinnati, O.

*New England Dental Society*—Meets annually. Next meeting 2nd Thursday in Nov., 1895, in Boston Mass. Pres. J. F. Adams, Worcester, N. H.; Sec. E. O. Kinsman, Cambridge, Mass.

*Northern Illinois Dental Society*—Meets annually. Next meeting at Aurora, October 17th, 1895. Pres. T. W. Beckwith, Sterling; Sec. J. W. Cormany, Mt. Carroll.

*Northern Indiana Dental Society*—Meets annually third Tuesday in October. Pres. E. J. Church; Sec. C. G Keekn.

*Odontological Society of Pennsylvania*—Meets on the first Saturday evening of each month, at 8 o'clock p. m., at 13th and Arch sts; Pres. Jas. Truman, Rec. Sec. A W. Deane.

*Odontological Society of New York City*—Meets the third Tuesday of each month. Pres. Dr. A. L. Northrop; Cor. Sec. Dr. George A. Wilson.

*Odontological Society of Cincinnati*—Meets monthly. Pres. G. Molyneaux; Sec. H. C. Matlack.

*Odontological Society of Chicago*—Meets third Tuesday each month. Pres. S. A. Swasey; Sce. and Treas., Louis Ottofy.

*Odontographic Society of Chicago*—Meets second Monday each month. Pres. Dr. M. Gallie; Sec. H. H. Wilson.

*Pennsylvania Association of Dental Surgeons*—Meets second Tuesday of each month. Pres. H. E. Roberts; Cor. Sec. Theo. F. Chupein.

*Pittsburg Dental Society*—Meets the last Tuesday Evening of each month. Pres. J. B. Williams, Homestead; Sec. N. L. Reinecke.

*Quincy, (Ills.) Dental Society*—Meets Monthly.

*San Francisco Dental Society*—Meets the second Monday evening of each month. Annual Meeting in October. Pres. C. E. Post; Sec. G. N. Van Orden; Cor. Sec. H. G. Richards.

*Sixth District Dental Society of N. Y.*—Meets semi-annually. The annual meeting will be held at Binghamton, the first week in May, 1895. Pres., M. H. Fish, New Berlin; Sec., F. W. McCall, Binghamton.

*Seventh District Dental Society of Ohio*—Meets annually. Next meeting in May, 1894. Pres., O. N. Heise, Cincinnati; Sec., J. R. Callahan, Cincinnati.

*Southern Illinois Dental Society*-Meets annually. Next annual meeting will be held on the 3d Tuesday in October, 1896. Pres., Sec., G. W. Entsminger, Carbondale.

*Southern Minnesota Dental Society*—Next meeting in Mankato, third Tuesday in April, 1895. Pres. S. Bond, Onoka; Sec. F. B. Kremer, Minneapolis.

*Stomatological Club of California*—Meets in Sanfrancisco. Pres. W. J. Younger.

*St. Louis Dental Society*—Meets first and third Tuesday in each month. Pres. J. B. Newby; Cor. Sec. John G Harper; Rec. Sec. F. F. Fletcher.

*The Isaac Knapp Dental Coterie of Fort Wayne, Ind.*—Meets the first Thursday of each month. Pres., W. W. Mungen; Sec., M. A. Mason.

*The Minneapolis Dental Society Monthly*—Pres. E. F. Clark, Sec. E. J. Morrison.

*The Tacoma Dental Society*—Meets monthly. Pres. M.C. Burns; Sec. W.E. Burkhart,

*Washington City Dental Society*—Meets 3d Tuesday of each month. Pres Dr. J. Roland Walton; Sec. Dr. D. Elmer Wiber.

### DIRECTORY OF DENTAL SOCIETIES.

*American Dental Association*—Meets first Tuesday in August, 1896. At Saratoga, N.Y. Pres., J. Y. Crawford, Nashville, Tenn.; Rec. Sec., Geo. H. Cushing, Chicago.

*Southern Dental Association*—Next meeting will be held at Nashville, Tenn., November, 1896. Pres., H. E. Beach, Clarksville, Tenn. Sec., S. W. Foster, Decatur, Ala.

### STATE DENTAL SOCIETIES.

*Alabama Dental Association*—Meets annually. Next meeting at Selma, on the second Tuesday of April, 1896. Pres. A. A. Pearson, Montgomery; Sec. S. W. Foster, Decatur.

*California State Dental Association*—Next meeting will be held in San Francisco, second Tuesday in June, 1895. Pres., L. A. Teague, San Francisco; Rec. Sec., W. Z. King, San Francisco; Cor. Sec., C. E. Post, San Francisco.

*Colorado State Dental Society*—Meets 1st Tuesday in June, 1894, at Glenwood Springs. Pres. P. T. Smith, Denver; Sec., Sarah May Townsend, Denver.

*Connecticut State Dental Society*—Next meeting will be held in Hartford, third Tuesday in May, 1895. Pres. Chas. P. Graham, Middletown; Rec. Sec. Geo. L. Parmele, Hartford.

*Dental Society of the State of Maryland and District of Columbia*—Meets annually. Next meeting at Washington City, on the second Tuesday in October, 1893. Pres. B. F. Coy, of Baltimore: Sec. M. W. Foster, Baltimore.

*Florida State Dental Association*—Meets annually. Next meeting at Tampa, 1st Tuesday in May, 1895. Pres, W. A. McQuaig, Lake City; Cor. Sec. S. W. Allen, Tampa.

*Georgia State Dental Society*—Meets at Indian Spring, in 1895. Exact time to be decided later; Pres. W. W. Hill, Washington; Rec. Sec. S. H. McKee, Americus; Cor. Sec. O. H. McDonald, Atlanta.

*Illinois State Dental Society*—The next meeting will be held at Springfield, Ill., May 12 to 15, 1896. Pres. W. A. Stevens, Chicago., Sec. Louis Ottofy, Chicago.

*Indiana State Dental Society*—Will meet at Detroit, Mich., on the third Tuesday of June, 1895. Pres. D. S. Harrold, Elwood; Secretary, G. E. Hunt, Indianapolis.

*Iowa State Dental Society*—Meets annually. Next meeting at Iowa City, May, 7th to 10th, inclusive, 1895. Pres. J. S. Kulp, Muscatine; Rec. Sec. F. T. Breene, Iowa City.

*Kentucky State Dental Association*—Meets annually. Next meeting in Louisville, Ky., 3rd Tuesday in June, 1896. Pres., J. H. Letcher, Danville; Sec., J. H. Baldwin, Louisville.

*Kansas State Dental Association*—Pres. C. B. Reed, Topeka; Sec., W. W. West, Topeka. Next meeting will be held at Topeka, second Monday in May, 1895.

*Maine Dental Society*—Will meet on the third Tuesday in July, 1895, in Bangor. Pres. T E. Tibbetts, Rockland; Sec. F. A. Knowlton, Fairfield.

*Maryland State Dental Association*—Next annual meeting will be held (place not definitely known yet,) in April, 1895. Pres., Chas. C. Harris, Baltimore; Sec, W. W. Dunbracco, Baltimore.

*Massachusetts Dental Society*—Meets annually in Boston, in June. Pres. Josiah W. Ball, Boston; Rec. Sec., E. O. Kinsman, Cambridge.

*Michigan State Dental Association*—Meets annually. Next meeting at Detroit, on the 3rd., Tuesday of June, 1896. Pres., J. Ward House, Grand Rapids; Sec. ———. Treas. Geo. H. Mosher, Jackson.

*Mississippi Dental Association*—Next meeting date to be fixed, 189[illegible], at Jackson, Miss. Pres. T. C. West, Natchez; Sec. L. N. Jeffries, Natchez.

*Minnesota State Society*—Pres. E. B. Weeks, Leitchfield; Sec., H. L. Cruttenden, St. Paul. Will meet in Winona, in September, 1896.

*Missouri Dental Association*—Meets at Bertle Springs, Mo., second Tuesday in July, 1895. Pres., J. T. Fry, Moberly; Sec., W. W. Carter, Sedalia.

*Nebraska State Dental Society*.—Next annual meeting will be held at Norfolk, third Tuesday in May, 1895. Pres., H. W. Shriver, Omaha; Cor. Sec., O. M. Huestis, Nebraska City.

*New Jersey State Dental Society*—Meets annually. The next meeting will be held August, 1896. Pres. R. M. Sanger, E. Orange; Sec. C. A. Meeker, Newark.

*New Hampshire Dental Society*—Meets Annually. Next meeting will be held in Concord, N. H.; time to be decided later. Pres. W. R. Blackstone, Manchester; Sec. Burton C. Russell, Keene.

*North Carolina State Dental Society*—Meets in Salisbury, on the second Tuesday in May, 1895. Pres. H. H. Harper, Kinston; Sec., J. E. Wyche, Greensboro.

*North Dakota State Dental Society*—The next meeting will be held at Fargo, May, 1895. Pres. E. M. Pierce, Hillsboro; Sec. C. L. Rose, Fargo.

*Ohio State Dental Society*—Meets in Columbus, first Tuesday in December, 1896. Pres. Hervey Barnes, Cleveland; Sec. L. P. Bethel, Kent.

*Pennsylvania State Dental Society*—Will hold its next annual meeting at Eagle's Mere, Pennsylvania, July 9th, 1895. Pres., Dr. J. A. Libbey, Pittsburgh; Cor. Sec., H. E. Roberts, Philadelphia.

*Rhode Island Dental Association*—Pres., C. M. Noble, Providence; Sec., H. W. Gillett, Newport, Meets quarterly, second Tuesday in July, October, January and April. Annual Meeting second Tuesday in July, at Newport.

*South Carolina State Dental Association*—Meets annually. Pres. J. C. Orland, Spartanburg; Cor. Sec. C. B. Colson, Charleston. Next meeting at Spartanburg, October, 1895.

*Tennessee Dental Association*—Meets annually. Next meeting at Knoxville, July 9, 1895. Pres. W. H. Richards, Knoxville; Rec. Sec. W. P. Houston, Lawrenceburg; Cor. Sec. W. J. Morrison, Nashville.

*The Dental Society of the State of New York*—Meets annually on the second Wednesday in May. Next session at Albany, 8th and 9th of May, 1895. Pres. F. T. Van Woert, Brooklyn; Sec. C. S. Butler, Buffalo; Cor. Sec. R. Ottolengui, New York.

*Texas State Dental Association*—Meets in San Antonia, third Tuesday in May, 1894. Pres. A. A. Beville, Waco; Sec. Chas. B. Lewis, Ennis

*Vermont State Dental Society*—Meets annually. Next meeting will be held at Brandon, Vt., third Wednesday in March, 1895. Pres. W. H. Wright, Brandon; Sec. Thos. Mound, Rutland.

*Virginia State Dental Association*—Will meet in 1895. Exact time and place to be decided later. Pres. H. W. Campbell, Suffolk; Sec. J. Hall Moore, Richmond.

*Washington State Dental Society*—Meets Annually. Next meeting will be held at Seattle, in the middle of May, 1895. Pres. B. S. Scott, Ellenburg; Sec., R. B. Gentle, Olympia.

*Wisconsin State Dental Society*—Meets annually. Next meeting will be held at Madison, Wis., the third Tuesday in July, 1895. Pres., Chas. C. Chittenden, Madison; Sec., C. A. Southwell, Milwaukee.

*West Virginia State Society.*—The annual meeting will be held at Parkersburg, on the first Wednesday of October, 1894. Pres. J. N. Mahan, Charleston; Sec. Geo. I. Keener, Morgantown.

### LOCAL SOCIETIES.

*American Academy of Dental Science*—Meets annually in Boston, Mass., on the first Wednesday in October. Pres. J. H. Batchelder, Rec. Sec. William Y. Allen.

*Atktnsonian Dental Society of Chicogo*—Meets second Tuesday each month. Pres. J. G. Reed; Sec. C. E. Meehoff.

*Buffalo Dental Association*—Meets last Monday evening of each month. Annual meeting in June. Pres. F. H. Boswell; Sec. J. A. Stackhouse.

*Brooklyn Dental Society and Second District Society United*—Meets quarterly—Pres. T. H. Holly; Rec. Sec. A. N. Russell, Brooklyn.

*Central Dental Association of Northern New Jersey*—Meets bi-monthly; and annually. The next annual meeting will be held about the middle of February, 1895. Pres. Thomas Moore, Paterson; Sec. Wm. L. Fish, Newark.

*Chicago Dental Society*—Meets monthly. Pres. W. V. B. Ames; Sec. A. H. Peck;

*Chicago Dental Club*—Meets on the fourth Monday of each month. Pres. I. D. Sperling; Sec. E. L. Clifford.

*Cleveland Dental Society.*—Meets the first Monday of each month. Pres. J. R. Bell; Sec. J. F. Stephan.

*Connecticut Valley Dental Society*—Meets annually. Next meeting in Oct. 1895, time and place to be decided later. Pres. C. C. Barker, Meriden, Conn.; Sec. Geo. A. Maxfield, Holyoke, Mass.

*Detroit Dental Society*—Meets monthly, Pres. Geo. L. Field, Sec. A. W. Diack.

*Dental Society of Southwestern Michigan*—Meets at St. Joseph, October 8th and 9th, 1895. Pres. S. M. White; Sec. E. I. Backers.

*Eighth District Dental Society, N. Y.*—Next annual meeting April 24th, 1894, in Buffalo. Pres. F. J. Burkhart, Batavia; Sec., W. V. Grove, Buffalo.

*East Tennessee Dental Association*—Meets annually. Next meeting will be held at Harriman, second Tuesday in June 1895. Pres. F. A. Shotwell, Rogersville; Sec. and Treas. Geo. C. Brause, Harriman.

*Eastern Illinois Dental Society*—Meets annually.

*Fifth District Dental Society of N. Y.*—Meets semi-annually. Next annual meeting will be held in Utica, 2d Tuesday in April, 1895. Pres. Frank Jones, Syracuse; Cor. Sec. A. R. Cooke, Syracuse.

*First District Dental Society of the State of New York*—Meets annually. The next meeting will be held on the second Tuesday of April 1895. Pres., Victor Hugo Jackson, New York City; Sec., Benj. C. Nash, New York City.

*First District Dental Society of Illinois*—Next meeting will be held in Canton, second Tuesday in September, 1895. Pres. W. A. Johnston, Peoria; Sec. W. O. Butter, La Harpe.

*Hayden Dental Society of Chicago*—Meets third Monday each month. Pres. T. E. Powell; Sec. A. J. Oakey.

*Knoxville City Dental Society*—Meets monthly. President, J. T. Cazier; Sec. A. J Cottrell.

*Minnesota State Dental Society*—Annual Meeting will be held in St. Paul, 2nd Wednesday in Sept., 1895. Pres. C. H. Robinsin, Wabasha; Sec. W. L. Cruttenden, Northfield.

*Mississippi Valley Dental Society*—Meets annually at Cincinnati. Next meeting March, 1896. Pres., W. V. B. Ames, Chicago; Rec. Sec. H. T. Smith, Cincinnati; Cor. Sec. H. C. Matlack, Cincinnati, O.

*New England Dental Society*—Meets annually. Next meeting 2nd Thursday in Nov., 1895, in Boston Mass. Pres. J. F. Adams, Worcester, N. H.; Sec. E. O. Kinsman, Cambridge, Mass.

*Northern Illinois Dental Society*—Meets annually. Next meeting at Aurora, October 17th, 1895. Pres. T. W. Beckwith, Sterling; Sec. J. W. Cormany, Mt. Carroll.

*Northern Indiana Dental Society*—Meets annually third Tuesday in October. Pres. E. J. Church; Sec. C. G Keekn.

*Odontological Society of Pennsylvania*—Meets on the first Saturday evening of each month, at 8 o'clock p. m., at 13th and Arch sts; Pres. Jas. Truman, Rec. Sec. A W. Deane.

*Odontological Society of New York City*—Meets the third Tuesday of each month. Pres. Dr. A. L. Northrop; Cor. Sec. Dr. George A. Wilson.

*Odontological Society of Cincinnati*—Meets monthly. Pres. G. Molyneaux; Sec. H. C. Matlack.

*Odontological Society of Chicago*—Meets third Tuesday each month. Pres. S. A. Swasey; Sce. and Treas., Louis Ottofy.

*Odontographic Society of Chicago*—Meets second Monday each month. Pres. Dr. M. Gallie; Sec. H. H. Wilson.

*Pennsylvania Association of Dental Surgeons*—Meets second Tuesday of each month. Pres. H. E. Roberts; Cor. Sec. Theo. F. Chupein.

*Pittsburg Dental Society*—Meets the last Tuesday Evening of each month. Pres. J. B. Williams, Homestead; Sec. N. L. Reinecke.

*Quincy, (Ills.) Dental Society*—Meets Monthly.

*San Francisco Dental Society*—Meets the second Monday evening of each month. Annual Meeting in October. Pres. C. E. Post; Sec. G. N. Van Orden; Cor. Sec. H. G. Richards.

*Sixth District Dental Society of N. Y.*—Meets semi-annually. The annual meeting will be held at Binghamton, the first week in May, 1895. Pres., M. H. Fish, New Berlin; Sec., F. W. McCall, Binghamton.

*Seventh District Dental Society of Ohio*—Meets annually. Next meeting in May, 1894. Pres., O. N. Heise, Cincinnati; Sec., J. R. Callahan, Cincinnati.

*Southern Illinois Dental Society*-Meets annually. Next annual meeting will be held on the 3d Tuesday in October, 1896. Pres., Sec., G. W. Entsminger, Carbondale.

*Southern Minnesota Dental Society*—Next meeting in Mankato, third Tuesday in April, 1895. Pres. S. Bond, Onoka: Sec. F. B. Kremer, Minneapolis.

*Stomatological Club of California*—Meets in Sanfrancisco. Pres. W. J. Younger.

*St. Louis Dental Society*—Meets first and third Tuesday in each month. Pres. J. B. Newby; Cor. Sec. John G Harper; Rec. Sec. F. F. Fletcher.

*The Isaac Knapp Dental Coterie of Fort Wayne, Ind.*—Meets the first Thursday of each month. Pres., W. W. Mungen; Sec., M. A. Mason.

*The Minneapolis Dental Society Monthly*—Pres. E. F. Clark, Sec. E. J. Morrison.

*The Tacoma Dental Society*—Meets monthly. Pres. M.C. Burns; Sec. W.E. Burkhart,

*Washington City Dental Society*—Meets 3d Tuesday of each month. Pres. Dr. J. Roland Walton; Sec. Dr. D. Elmer Wiber.

*North Carolina State Dental Society*—Meets in Salisbury, on the second Tuesday in May, 1895. Pres. H. H. Harper, Kinston; Sec., J. E. Wyche, Greensboro.

*North Dakota State Dental Society*—The next meeting will be held at Fargo, May, 1895. Pres. E. M. Pierce, Hillsboro; Sec. C. L. Rose, Fargo.

*Ohio State Dental Society*—Meets in Columbus, first Tuesday in December, 1896. Pres. Hervey Barnes, Cleveland; Sec. L. P. Bethel, Kent.

*Pennsylvania State Dental Society*—Will hold its next annual meeting at Eagle's Mere, Pennsylvania, July 9th, 1895. Pres., Dr. J. A. Libbey, Pittsburgh; Cor. Sec., H. E. Roberts, Philadelphia.

*Rhode Island Dental Association*—Pres., C. M. Noble, Providence; Sec., H. W. Gillett, Newport, Meets quarterly, second Tuesday in July, October, January and April. Annual Meeting second Tuesday in July, at Newport.

*South Carolina State Dental Association*—Meets annually. Pres. J. C. Orland, Spartanburg; Cor. Sec. C. B. Colson, Charleston. Next meeting at Spartanburg, October, 1895.

*Tennessee Dental Association*—Meets annually. Next meeting at Knoxville, July 9, 1895. Pres. W. H. Richards, Knoxville; Rec Sec. W. P. Houston, Lawrenceburg; Cor. Sec. W. J. Morrison, Nashville.

*The Dental Society of the State of New York*—Meets annually on the second Wednesday in May. Next session at Albany, 8th and 9th of May, 1895. Pres. F. T. Van Woert, Brooklyn; Sec. C. S. Butler, Buffalo; Cor. Sec. R. Ottolengui, New York.

*Texas State Dental Association*—Meets in San Antonia, third Tuesday in May, 1894. Pres. A. A. Beville, Waco; Sec. Chas. B. Lewis, Ennis

*Vermont State Dental Society*—Meets annually. Next meeting will be held at Brandon, Vt., third Wednesday in March, 1895. Pres. W. H. Wright, Brandon; Sec. Thos. Mound, Rutland.

*Virginia State Dental Association*—Will meet in 1895. Exact time and place to be decided later. Pres. H. W. Campbell, Suffolk; Sec. J. Hall Moore, Richmond.

*Washington State Dental Society*—Meets Annually. Next meeting will be held at Seattle, in the middle of May, 1895. Pres. B. S. Scott, Ellenburg; Sec., R. B. Gentle, Olympia.

*Wisconsin State Dental Society*—Meets annually. Next meeting will be held at Madison, Wis., the third Tuesday in July, 1895. Pres., Chas. C. Chittenden, Madison; Sec., C. A. Southwell, Milwaukee.

*West Virginia State Society.*—The annual meeting will be held at Parkersburg, on the first Wednesday of October, 1894. Pres. J. N. Mahan, Charleston; Sec. Geo. I. Keener, Morgantown.

### LOCAL SOCIETIES.

*American Academy of Dental Science*—Meets annually in Boston, Mass., on the first Wednesday in October. Pres. J. H. Batchelder, Rec. Sec. William Y. Allen.

*Atktnsonian Dental Society of Chicogo*—Meets second Tuesday each month. Pres. J. G. Reed; Sec. C. E. Meehoff.

*Buffalo Dental Association*—Meets last Monday evening of each month. Annual meeting in June. Pres. F. H. Boswell; Sec. J. A. Stackhouse.

*Brooklyn Dental Society and Second District Society United*—Meets quarterly—Pres. T. H. Holly; Rec. Sec. A. N. Russell, Brooklyn.

*Central Dental Association of Northern New Jersey*—Meets bi-monthly; and annually. The next annual meeting will be held about the middle of February, 1895. Pres. Thomas Moore, Paterson; Sec. Wm. L. Fish, Newark.

*Chicago Dental Society*—Meets monthly. Pres. W. V. B. Ames; Sec. A. H. Peck;

*Chicago Dental Club*—Meets on the fourth Monday of each month. Pres. I. D. Sperling; Sec. E. L. Clifford.

*Cleveland Dental Society.*—Meets the first Monday of each month. Pres. J. R. Bell; Sec. J. F. Stephan.

*Connecticut Valley Dental Society*—Meets annually. Next meeting in Oct. 1895, time and place to be decided later. Pres. C. C. Barker, Meriden, Conn.; Sec. Geo. A. Maxfield, Holyoke, Mass.

*Detroit Dental Society*—Meets monthly, Pres. Geo. L. Field, Sec. A. W. Diack.

*Dental Society of Southwestern Michigan*—Meets at St. Joseph, October 8th and 9th, 1895. Pres. S. M. White; Sec. E. I. Backers.

*Eighth District Dental Society, N. Y.*—Next annual meeting April 24th, 1894, in Buffalo. Pres. F. J. Burkhart, Batavia; Sec., W. V. Grove, Buffalo.

*East Tennessee Dental Association*—Meets annually. Next meeting will be held at Harriman, second Tuesday in June 1895. Pres. F. A. Shotwell, Rogersville; Sec. and Treas. Geo. C. Brause, Harriman.

### DIRECTORY OF DENTAL SOCIETIES.

*American Dental Association*—Meets first Tuesday in August, 1896. At Saratoga, N.Y. Pres., J. Y. Crawford, Nashville, Tenn.; Rec. Sec., Geo. H. Cushing, Chicago.

*Southern Dental Association*—Next meeting will be held at Nashville, Tenn., November, 1896. Pres., H. E. Beach, Clarksville, Tenn. Sec., S. W. Foster, Decatur, Ala.

### STATE DENTAL SOCIETIES.

*Alabama Dental Association*—Meets annually. Next meeting at Selma, on the second Tuesday of April, 1896. Pres. A. A. Pearson, Montgomery; Sec. S. W. Foster, Decatur.

*California State Dental Association*—Next meeting will be held in San Francisco, second Tuesday in June, 1895. Pres., L. A. Teague, San Francisco; Rec. Sec., W. Z. King, San Francisco; Cor. Sec., C. E. Post, San Francisco.

*Colorado State Dental Society*—Meets 1st Tuesday in June, 1894, at Glenwood Springs. Pres. P. T. Smith, Denver; Sec., Sarah May Townsend, Denver.

*Connecticut State Dental Society*—Next meeting will be held in Hartford, third Tuesday in May, 1895. Pres. Chas. P. Graham, Middletown; Rec. Sec. Geo. L. Parmele, Hartford.

*Dental Society of the State of Maryland and District of Columbia*—Meets annually. Next meeting at Washington City, on the second Tuesday in October, 1893. Pres. B. F. Coy, of Baltimore: Sec. M. W. Foster, Baltimore.

*Florida State Dental Association*—Meets annually. Next meeting at Tampa, 1st Tuesday in May, 1895. Pres, W. A. McQuaig, Lake City; Cor. Sec. S. W. Allen, Tampa.

*Georgia State Dental Society*—Meets at Indian Spring, in 1895. Exact time to be decided later; Pres. W. W. Hill, Washington; Rec. Sec. S. H. McKee, Americus; Cor. Sec. O. H. McDonald, Atlanta.

*Illinois State Dental Society*—The next meeting will be held at Springfield, Ill., May 12 to 15, 1896. Pres. W. A. Stevens, Chicago., Sec. Louis Ottofy, Chicago.

*Indiana State Dental Society*—Will meet at Detroit, Mich., on the third Tuesday of June, 1895. Pres. D. S. Harrold, Elwood; Secretary, G. E. Hunt, Indianapolis.

*Iowa State Dental Society*—Meets annually. Next meeting at Iowa City, May, 7th to 10th, inclusive, 1895. Pres. J. S. Kulp, Muscatine; Rec. Sec. F. T. Breene, Iowa City.

*Kentucky State Dental Association*—Meets annually. Next meeting in Louisville, Ky., 3rd Tuesday in June, 1896. Pres., J. H. Letcher, Danville; Sec., J. H. Baldwin, Louisville.

*Kansas State Dental Association*—Pres. C. B. Reed, Topeka; Sec., W. W. West, Topeka. Next meeting will be held at Topeka, second Monday in May, 1895.

*Maine Dental Society*—Will meet on the third Tuesday in July, 1895, in Bangor. Pres. T E. Tibbetts, Rockland; Sec. F. A. Knowlton, Fairfield.

*Maryland State Dental Association*—Next annual meeting will be held (place not definitely known yet,) in April, 1895. Pres., Chas. C. Harris, Baltimore; Sec, W. W. Dunbracco, Baltimore.

*Massachusetts Dental Society*—Meets annually in Boston, in June. Pres. Josiah W. Ball, Boston; Rec. Sec., E. O. Kinsman, Cambridge.

*Michigan State Dental Association*—Meets annually. Next meeting at Detroit, on the 3rd., Tuesday of June, 1896. Pres., J. Ward House, Grand Rapids; Sec. ————. Treas. Geo. H. Mosher, Jackson.

*Mississippi Dental Association*—Next meeting date to be fixed, 1896, at Jackson, Miss. Pres. T. C. West, Natchez; Sec. L. N. Jeffries, Natchez.

*Minnesota State Society*—Pres. E. B. Weeks, Leitchfield; Sec., H. L. Cruttenden, St. Paul. Will meet in Winona, in September, 1896.

*Missouri Dental Association*—Meets at Bertle Springs, Mo., second Tuesday in July, 1895. Pres., J. T. Fry, Moberly; Sec., W. W. Carter, Sedalia.

*Nebraska State Dental Society.*—Next annual meeting will be held at Norfolk, third Tuesday in May, 1895. Pres., H. W. Shriver, Omaha; Cor. Sec., O. M. Huestis, Nebraska City.

*New Jersey State Dental Society*—Meets annually. The next meeting will be held August, 1896. Pres. R. M. Sanger, E. Orange; Sec. C. A. Meeker, Newark.

*New Hampshire Dental Society*—Meets Annually. Next meeting will be held in Concord, N. H.; time to be decided later. Pres. W. R. Blackstone, Manchester; Sec. Burton C. Russell, Keene.

*Eastern Illinois Dental Society*—Meets annually.

*Fifth District Dental Society of N. Y.*—Meets semi-annually. Next annual meeting will be held in Utica, 2d Tuesday in April, 1895. Pres. Frank Jones, Syracuse; Cor. Sec. A. R. Cooke, Syracuse.

*First District Dental Society of the State of New York*—Meets annually. The next meeting will be held on the second Tuesday of April 1895. Pres., Victor Hugo Jackson, New York City; Sec., Benj. C. Nash, New York City.

*First District Dental Society of Illinois*—Next meeting will be held in Canton, second Tuesday in September, 1895. Pres. W. A. Johnston, Peoria; Sec. W. O. Butter, La Harpe.

*Hayden Dental Society of Chicago*—Meets third Monday each month. Pres. T. E. Powell; Sec. A. J. Oakey.

*Knoxville City Dental Society*—Meets monthly. President, J. T. Cazier; Sec. A. J. Cottrell.

*Minnesota State Dental Society*—Annual Meeting will be held in St. Paul, 2nd Wednesday in Sept., 1895. Pres. C. H. Robinsin, Wabasha; Sec. W. L. Cruttenden, Northfield.

*Mississippi Valley Dental Society*—Meets annually at Cincinnati. Next meeting March, 1896. Pres., W. V. B. Ames, Chicago; Rec. Sec. H. T. Smith, Cincinnati; Cor. Sec. H. C. Matlack, Cincinnati, O.

*New England Dental Society*—Meets annually. Next meeting 2nd Thursday in Nov., 1895, in Boston Mass. Pres. J. F. Adams, Worcester, N. H.; Sec. E. O. Kinsman, Cambridge, Mass.

*Northern Illinois Dental Society*—Meets annually. Next meeting at Aurora, October 17th, 1895. Pres. T. W. Beckwith, Sterling; Sec. J. W. Cormany, Mt. Carroll.

*Northern Indiana Dental Society*—Meets annually third Tuesday in October. Pres. E. J. Church; Sec. C. G Keekn.

*Odontological Society of Pennsylvania*—Meets on the first Saturday evening of each month, at 8 o'clock p. m., at 13th and Arch sts; Pres. Jas. Truman, Rec. Sec. A W. Deane.

*Odontological Society of New York City*—Meets the third Tuesday of each mont[illegible]. Pres. Dr. A. L. Northrop; Cor. Sec. Dr. George A. Wilson.

*Odontological Society of Cincinnati*—Meets monthly. Pres. G. Molyneaux; Sec. H. C. Matlack.

*Odontological Society of Chicago*—Meets third Tuesday each month. Pres. S. A. Swasey; Sce. and Treas., Louis Ottofy.

*Odontographic Society of Chicago*—Meets second Monday each month. Pres. Dr. M. Gallie; Sec. H. H. Wilson.

*Pennsylvania Association of Dental Surgeons*—Meets second Tuesday of each month. Pres. H. E. Roberts; Cor. Se[illegible]. Theo. F. Chupein.

*Pittsburg Dental Society*—Meets the last Tuesday Evening of each month. Pres. J. B. Williams, Homestead; Sec. N. L. Reinecke.

*Quincy, (Ills.) Dental Society*—Meets Monthly.

*San Francisco Dental Society*—Meets the second Monday evening of each month. Annual Meeting in October. Pres. C. E. Post; Sec. G. N. Van Orden; Cor. Sec. H. G. Richards.

*Sixth District Dental Society of N. Y.*—Meets semi-annually. The annual meeting will be held at Binghamton, the first week in May, 1895. Pres., M. H. Fish, New Berlin; Sec., F. W. McCall, Binghamton.

*Seventh District Dental Society of Ohio*—Meets annually. Next meeting in May, 1894. Pres., O. N. Heise, Cincinnati; Sec., J. R. Callahan, Cincinnati.

*Southern Illinois Dental Society*—Meets annually. Next annual meeting will be held on the 3d Tuesday in October, 1896. Pres., Sec., G. W. Entsminger, Carbondale.

*Southern Minnesota Dental Society*—Next meeting in Mankato, third Tuesday in April, 1895. Pres. S. Bond, Onoka: Sec. F. B. Kremer, Minneapolis.

*Stomatological Club of California*—Meets in Sanfrancisco. Pres. W. J. Younger.

*St. Louis Dental Society*—Meets first and third Tuesday in each month. Pres. J. B. Newby; Cor. Sec. John G Harper; Rec. Sec. F. F. Fletcher.

*The Isaac Knapp Dental Coterie of Fort Wayne, Ind.*—Meets the first Thursday of each month. Pres., W. W. Mungen; Sec., M. A. Mason.

*The Minneapolis Dental Society Monthly*—Pres. E. F. Clark, Sec. E. J. Morrison.

*The Tacoma Dental Society*—Meets monthly. Pres. M.C. Burns; Sec. W.E. Burkhart,

*Washington City Dental Society*—Meets 3d Tuesday of each month. Pres. Dr. J. Roland Walton; Sec. Dr. D. Elmer Wiber.

### DIRECTORY OF DENTAL SOCIETIES.

*American Dental Association*—Meets first Tuesday in August, 1896. At Saratoga, N.Y. Pres., J. Y. Crawford, Nashville, Tenn.; Rec. Sec., Geo. H. Cushing, Chicago.

*Southern Dental Association*—Next meeting will be held at Nashville, Tenn., November, 1896. Pres., H. E. Beach, Clarksville, Tenn. Sec., S. W. Foster, Decatur, Ala.

### STATE DENTAL SOCIETIES.

*Alabama Dental Association*—Meets annually. Next meeting at Selma, on the second Tuesday of April, 1896. Pres. A. A. Pearson, Montgomery; Sec. S. W. Foster, Decatur.

*California State Dental Association*—Next meeting will be held in San Francisco, second Tuesday in June, 1896. Pres., L. A. Teague, San Francisco; Rec. Sec., W. Z. King, San Francisco; Cor. Sec., C. E. Post, San Francisco.

*Colorado State Dental Society*—Meets 1st Tuesday in June, 1894, at Glenwood Springs. Pres. P. T. Smith, Denver; Sec., Sarah May Townsend, Denver.

*Connecticut State Dental Society*—Next meeting will be held in Hartford, third Tuesday in May, 1895. Pres. Chas. P. Graham, Middletown; Rec. Sec. Geo. L. Parmele, Hartford.

*Dental Society of the State of Maryland and District of Columbia*—Meets annually. Next meeting at Washington City, on the second Tuesday in October, 1893. Pres. B. F. Coy. of Baltimore: Sec. M. W. Foster. Baltimore.

*Florida State Dental Association*—Meets annually. Next meeting at Tampa, 1st Tuesday in May, 1895. Pres, W. A. McQuaig, Lake City; Cor. Sec. S. W. Allen, Tampa.

*Georgia State Dental Society*—Meets at Indian Spring, in 1895. Exact time to be decided later; Pres. W. W. Hill, Washington; Rec. Sec. S. H. McKee, Americus; Cor. Sec. O. H. McDonald, Atlanta.

*Illinois State Dental Society*—The next meeting will be held at Springfield, Ill., May 12 to 15, 1896. Pres. W. A. Stevens, Chicago., Sec. Louis Ottofy, Chicago.

*Indiana State Dental Society*—Will meet at Detroit, Mich., on the third Tuesday of June, 1895. Pres. D. S. Harrold, Elwood; Secretary, G. E. Hunt, Indianapolis.

*Iowa State Dental Society*—Meets annually. Next meeting at Iowa City, May, 7th to 10th, inclusive, 1895. Pres. J. S. Kulp, Muscatine; Rec. Sec. F. T. Breene, Iowa City.

*Kentucky State Dental Association*—Meets annually. Next meeting in Louisville, Ky., 3rd Tuesday in June, 1896. Pres., J. H. Letcher, Danville; Sec., J. H. Baldwin, Louisville.

*Kansas State Dental Association*—Pres. C. B. Reed, Topeka; Sec., W. W. West, Topeka. Next meeting will be held at Topeka, second Monday in May, 1895.

*Maine Dental Society*—Will meet on the third Tuesday in July, 1895, in Bangor. Pres. T E. Tibbetts, Rockland; Sec. F. A. Knowlton, Fairfield.

*Maryland State Dental Association*—Next annual meeting will be held (place not definitely known yet,) in April, 1895. Pres., Chas. C. Harris, Baltimore; Sec, W. W. Dunbracco, Baltimore.

*Massachusetts Dental Society*—Meets annually in Boston, in June. Pres. Josiah W. Ball, Boston; Rec. Sec., E. O. Kinsman, Cambridge.

*Michigan State Dental Association*—Meets annually. Next meeting at Detroit, on the 3rd., Tuesday of June, 1896. Pres., J. Ward House, Grand Rapids; Sec. ————. Treas. Geo. H. Mosher, Jackson.

*Mississippi Dental Association*—Next meeting date to be fixed, 1895, at Jackson, Miss. Pres. T. C. West, Natchez; Sec. L. N. Jeffries, Natchez.

*Minnesota State Society*—Pres. E. B. Weeks, Leitchfield; Sec., H. L. Cruttenden, St. Paul. Will meet in Winona, in September, 1896.

*Missouri Dental Association*—Meets at Bertle Springs, Mo., second Tuesday in July, 1895. Pres., J. T. Fry, Moberly; Sec., W. W. Carter, Sedalia.

*Nebraska State Dental Society.*—Next annual meeting will be held at Norfolk, third Tuesday in May, 1895. Pres., H. W. Shriver, Omaha; Cor. Sec., O. M. Huestis, Nebraska City.

*New Jersey State Dental Society*—Meets annually. The next meeting will be held August, 1896. Pres. R. M. Sanger, E. Orange; Sec. C. A. Meeker, Newark.

*New Hampshire Dental Society*—Meets Annually. Next meeting will be held in Concord, N. H.; time to be decided later. Pres. W. R. Blackstone, Manchester; Sec. Burton C. Russell, Keene.

*North Carolina State Dental Society*—Meets in Salisbury, on the second Tuesday in May, 1895. Pres. H. H. Harper, Kinston; Sec., J. E. Wyche, Greensboro.

*North Dakota State Dental Society*—The next meeting will be held at Fargo, May, 1895. Pres. E. M. Pierce, Hillsboro; Sec. C. L. Rose, Fargo.

*Ohio State Dental Society*—Meets in Columbus, first Tuesday in December, 1896. Pres. Hervey Barnes, Cleveland; Sec. L. P. Bethel, Kent.

*Pennsylvania State Dental Society*—Will hold its next annual meeting at Eagle's Mere, Pennsylvania, July 9th, 1895. Pres., Dr. J. A. Libbey, Pittsburgh; Cor. Sec., H. E. Roberts, Philadelphia.

*Rhode Island Dental Association*—Pres., C. M. Noble, Providence; Sec., H. W. Gillett, Newport, Meets quarterly, second Tuesday in July, October, January and April. Annual Meeting second Tuesday in July, at Newport.

*South Carolina State Dental Association*—Meets annually. Pres. J. C. Orland, Spartanburg; Cor. Sec. C. B. Colson, Charleston. Next meeting at Spartanburg, October, 1895.

*Tennessee Dental Association*—Meets annually. Next meeting at Knoxville, July 9, 1895. Pres. W. H. Richards, Knoxville: Rec. Sec. W. P. Houston, Lawrenceburg; Cor. Sec. W. J. Morrison, Nashville.

*The Dental Society of the State of New York*—Meets annually on the second Wednesday in May. Next session at Albany, 8th and 9th of May, 1895. Pres. F. T. Van Woert, Brooklyn; Sec. C. S. Butler, Buffalo; Cor. Sec. R. Ottolengui, New York.

*Texas State Dental Association*—Meets in San Antonia, third Tuesday in May, 1894. Pres. A. A. Beville, Waco; Sec. Chas. B. Lewis, Ennis

*Vermont State Dental Society*—Meets annually. Next meeting will be held at Brandon, Vt., third Wednesday in March, 1895. Pres. W. H. Wright, Brandon; Sec. Thos. Mound, Rutland.

*Virginia State Dental Association*—Will meet in 1895. Exact time and place to be decided later. Pres. H. W. Campbell, Suffolk; Sec. J. Hall Moore, Richmond.

*Washington State Dental Society*—Meets Annually. Next meeting will be held at Seattle, in the middle of May, 1895. Pres. B. S. Scott, Ellenburg; Sec., R. B. Gentle, Olympia.

*Wisconsin State Dental Society*—Meets annually. Next meeting will be held at Madison, Wis., the third Tuesday in July, 1895. Pres., Chas. C. Chittenden, Madison; Sec., C. A. Southwell, Milwaukee.

*West Virginia State Society.*—The annual meeting will be held at Parkersburg, on the first Wednesday of October, 1894. Pres. J. N. Mahan, Charleston; Sec. Geo. I. Keener, Morgantown.

### LOCAL SOCIETIES.

*American Academy of Dental Science*—Meets annually in Boston, Mass., on the first Wednesday in October. Pres. J. H. Batchelder, Rec. Sec. William Y. Allen.

*Atktnsonian Dental Society of Chicogo*—Meets second Tuesday each month. Pres. J. G. Reed; Sec. C. E. Meehoff.

*Buffalo Dental Association*—Meets last Monday evening of each month. Annual meeting in June. Pres. F. H. Boswell; Sec. J. A. Stackhouse.

*Brooklyn Dental Society and Second District Society United*—Meets quarterly—Pres. T. H. Holly; Rec. Sec. A. N. Russell, Brooklyn.

*Central Dental Association of Northern New Jersey*—Meets bi-monthly; and annually. The next annual meeting will be held about the middle of February, 1895. Pres. Thomas Moore, Paterson; Sec. Wm. L. Fish, Newark.

*Chicago Dental Society*—Meets monthly. Pres. W. V. B. Ames; Sec. A. H. Peck;

*Chicago Dental Club*—Meets on the fourth Monday of each month. Pres. I. D. Sperling; Sec. E. L. Clifford.

*Cleveland Dental Society.*—Meets the first Monday of each month. Pres. J. R. Bell; Sec. J. F. Stephan.

*Connecticut Valley Dental Society*—Meets annually. Next meeting in Oct. 1895, time and place to be decided later. Pres. C. C. Barker, Meriden, Conn.; Sec. Geo. A. Maxfield, Holyoke, Mass.

*Detroit Dental Society*—Meets monthly, Pres. Geo. L. Field, Sec. A. W. Diack.

*Dental Society of Southwestern Michigan*—Meets at St. Joseph, October 8th and 9th, 1895. Pres. S. M. White; Sec. E. I. Backers.

*Eighth District Dental Society, N. Y.*—Next annual meeting April 24th, 1894, in Buffalo. Pres. F. J. Burkhart, Batavia; Sec., W. V. Grove, Buffalo.

*East Tennessee Dental Association*—Meets annually. Next meeting will be held at Harriman, second Tuesday in June 1895. Pres. F. A. Shotwell, Rogersville; Sec. and Treas. Geo. C. Brause, Harriman.

*Eastern Illinois Dental Society*—Meets annually.

*Fifth District Dental Society of N. Y.*—Meets semi-annually. Next annual meeting will be held in Utica, 2d Tuesday in April, 1895. Pres. Frank Jones, Syracuse; Cor. Sec. A. R. Cooke, Syracuse.

*First District Dental Society of the State of New York*—Meets annually. The next meeting will be held on the second Tuesday of April 1895. Pres., Victor Hugo Jackson, New York City; Sec., Benj. C. Nash, New York City.

*First District Dental Society of Illinois*—Next meeting will be held in Canton, second Tuesday in September, 1895. Pres. W. A. Johnston, Peoria; Sec. W. O. Butter, La Harpe.

*Hayden Dental Society of Chicago*—Meets third Monday each month. Pres. T. E. Powell; Sec. A. J. Oakey.

*Knoxville City Dental Society*—Meets monthly. President, J. T. Cazier; Sec. A. J. Cottrell.

*Minnesota State Dental Society*—Annual Meeting will be held in St. Paul, 2nd Wednesday in Sept., 1895. Pres. C. H. Robinsin, Wabasha; Sec. W. L. Cruttenden, Northfield.

*Mississippi Valley Dental Society*—Meets annually at Cincinnati. Next meeting March, 1896 Pres., Junicus E. Cravens, Indianapolis; Rec. Sec. H. T. Smith, Cincinnati; Cor. Sec. H. C. Matlack, Cincinnati, O.

*New England Dental Society*—Meets annually. Next meeting 2nd Thursday in Nov., 1895, in Boston Mass. Pres. J. F. Adams, Worcester, N. H.; Sec. E. O. Kinsman, Cambridge, Mass.

*Northern Illinois Dental Society*—Meets annually. Next meeting at Aurora, October 17th, 1895. Pres. T. W. Beckwith, Sterling; Sec. J. W. Cormany, Mt. Carroll.

*Northern Indiana Dental Society*—Meets annually third Tuesday in October. Pres. E. J. Church; Sec. C. G Keekn.

*Odontological Society of Pennsylvania*—Meets on the first Saturday evening of each month, at 8 o'clock p. m., at 13th and Arch sts; Pres. Jas. Truman, Rec. Sec. A W. Deane.

*Odontological Society of New York City*—Meets the third Tuesday of each month. Pres. Dr. A. L. Northrop; Cor. Sec. Dr. George A. Wilson.

*Odontological Society of Cincinnati*—Meets monthly. Pres. G. Molyneaux; Sec. H. C. Matlack.

*Odontological Society of Chicago*—Meets third Tuesday each month. Pres. S. A. Swasey; Sce. and Treas., Louis Ottofy.

*Odontographic Society of Chicago*—Meets second Monday each month. Pres. Dr. M. Gallie; Sec. H. H. Wilson.

*Pennsylvania Association of Dental Surgeons*—Meets second Tuesday of each month. Pres. H. E. Roberts; Cor. Se . Theo. F. Chupein.

*Pittsburg Dental Society*—Meets the last Tuesday Evening of each month. Pres. J. B. Williams, Homestead; Sec. N. L. Reinecke.

*Quincy, (Ills.) Dental Society*—Meets Monthly.

*San Francisco Dental Society*—Meets the second Monday evening of each month. Annual Meeting in October. Pres. C. E. Post; Sec. G. N. Van Orden; Cor. Sec. H. G. Richards.

*Sixth District Dental Society of N. Y.*—Meets semi-annually. The annual meeting will be held at Binghamton, the first week in May. 1895. Pres., M. H. Fish, New Berlin; Sec., F. W. McCall, Binghamton.

*Seventh District Dental Society of Ohio*—Meets annually. Next meeting in May, 1894. Pres., O. N. Heise, Cincinnati; Sec., J. R. Callahan, Cincinnati.

*Southern Illinois Dental Society* Meets annually. Next annual meeting will be held on the 3d Tuesday in October, 1896. Pres., Sec., G. W. Entsminger, Carbondale.

*Southern Minnesota Dental Society*—Next meeting in Mankato, third Tuesday in April, 1895. Pres. S. Bond, Onoka; Sec. F. B. Kremer, Minneapolis.

*Stomatological Club of California*—Meets in Sanfrancisco. Pres. W. J. Younger.

*St. Louis Dental Society*—Meets first and third Tuesday in each month. Pres. J. B. Newby; Cor. Sec. John G Harper; Rec. Sec. F. F. Fletcher.

*The Isaac Knapp Dental Coterie of Fort Wayne, Ind.*—Meets the first Thursday of each month. Pres., W. W. Mungen; Sec., M. A. Mason.

*The Minneapolis Dental Society Monthly*—Pres. E. F. Clark, Sec. E. J. Morrison.

*The Tacoma Dental Society*—Meets monthly. Pres. M.C. Burns; Sec.W.E.Burkhart

*Washington City Dental Society*—Meets 3d Tuesday of each month. Pres. Dr. J. Roland Walton; Sec. Dr. D. Elmer Wiber.

### DIRECTORY OF DENTAL SOCIETIES.

*American Dental Association*—Meets first Tuesday in August, 1896. At Saratoga, N.Y. Pres., J. Y. Crawford, Nashville, Tenn.; Rec. Sec., Geo. H. Cushing, Chicago.

*Southern Dental Association*—Next meeting will be held at Nashville, Tenn., November, 1896. Pres., H. E. Beach, Clarksville, Tenn. Sec., S. W. Foster, Decatur, Ala.

### STATE DENTAL SOCIETIES.

*Alabama Dental Association*—Meets annually. Next meeting at Selma, on the second Tuesday of April, 1896. Pres. A. A. Pearson, Montgomery; Sec. S. W. Foster, Decatur.

*California State Dental Association*—Next meeting will be held in San Francisco, second Tuesday in June, 1896. Pres., L. A. Teague, San Francisco; Rec. Sec., W. Z. King, San Francisco; Cor. Sec., C. E. Post, San Francisco.

*Colorado State Dental Society*—Meets 1st Tuesday in June, 1894, at Glenwood Springs. Pres. P. T. Smith, Denver; Sec., Sarah May Townsend, Denver.

*Connecticut State Dental Society*—Next meeting will be held in Hartford, third Tuesday in May, 1895. Pres. Chas. P. Graham, Middletown; Rec. Sec. Geo. L. Parmele, Hartford.

*Dental Society of the State of Maryland and District of Columbia*—Meets annually. Next meeting at Washington City, on the second Tuesday in October, 1893. Pres. B. F. Coy, of Baltimore: Sec. M. W. Foster, Baltimore.

*Florida State Dental Association*—Meets annually. Next meeting at Tampa, 1st Tuesday in May, 1895. Pres, W. A. McQuaig, Lake City; Cor. Sec. S. W. Allen, Tampa.

*Georgia State Dental Society*—Meets at Indian Spring, in 1895. Exact time to be decided later; Pres. W. W. Hill, Washington; Rec. Sec. S. H. McKee, Americus; Cor. Sec. O. H. McDonald, Atlanta.

*Illinois State Dental Society*—The next meeting will be held at Peoria, Ill., May Second Tuesday, 1897. Pres. C. R. Taylor, Streaton., Sec. Louis Ottofy, Chicago.

*Indiana State Dental Society*—Will meet at Detroit, Mich., on the third Tuesday of June, 1895. Pres. D. S. Harrold, Elwood; Secretary, G. E. Hunt, Indianapolis.

*Iowa State Dental Society*—Meets annually. Next meeting at Iowa City, May, 7th to 10tn, inclusive, 1895. Pres. J. S. Kulp, Muscatine; Rec. Sec. F. T. Breene, Iowa City.

*Kentucky State Dental Association*—Meets annually. Next meeting in Louisville, Ky., 3rd Tuesday in June, 1896. Pres., J. H. Letcher, Danville; Sec., J. H. Baldwin, Louisville.

*Kansas State Dental Association*—Pres. C. B. Reed, Topeka; Sec., W. W. West, Topeka. Next meeting will be held at Topeka, second Monday in May, 1895.

*Maine Dental Society*—Will meet on the third Tuesday in July, 1895, in Bangor. Pres. T E. Tibbetts, Rockland; Sec. F. A. Knowlton, Fairfield.

*Maryland State Dental Association*—Next annual meeting will be held (place not definitely known yet,) in April, 1895. Pres., Chas. C. Harris, Baltimore; Sec, W. W. Dunbracco, Baltimore.

*Massachusetts Dental Society*—Meets annually in Boston, in June. Pres. Josiah W. Ball, Boston; Rec. Sec., E. O. Kinsman, Cambridge.

*Michigan State Dental Association*—Meets annually. Next meeting at Detroit, on the 3rd., Tuesday of June, 1896. Pres., J. Ward House, Grand Rapids; Sec. ————. Treas. Geo. H. Mosher, Jackson.

*Mississippi Dental Association*—Next meeting date to be fixed, 1896, at Jackson, Miss. Pres. T. C. West, Natchez; Sec. L. N. Jeffries, Natchez.

*Minnesota State Society*—Pres. E. B. Weeks, Leitchfield; Sec., H. L. Cruttenden, St. Paul. Will meet in Winona, in September, 1896.

*Missouri Dental Association*—Meets at Bertle Springs, Mo., second Tuesday in July, 1895. Pres., J. T. Fry, Moberly; Sec., W. W. Carter, Sedalia.

*Nebraska State Dental Society.*—Next annual meeting will be held at Norfolk, third Tuesday in May, 1895. Pres., H. W. Shriver, Omaha; Cor. Sec., O. M. Huestis, Nebraska City.

*New Jersey State Dental Society*—Meets annually. The next meeting will be held August, 1896. Pres. R. M. Sanger, E. Orange; Sec. C. A. Meeker, Newark.

*New Hampshire Dental Society*—Meets Annually. Next meeting will be held in Concord, N. H.; time to be decided later. Pres. W. R. Blackstone, Manchester; Sec. Burton C. Russell, Keene.

*North Carolina State Dental Society*—Meets in Salisbury, on the second Tuesday in May, 1895. Pres. H. H. Harper, Kinston; Sec., J. E. Wyche, Greensboro.

*North Dakota State Dental Society*—The next meeting will be held at Fargo, May, 1895. Pres. E. M. Pierce, Hillsboro; Sec. C. L. Rose, Fargo.

*Ohio State Dental Society*—Meets in Columbus, first Tuesday in December, 1896. Pres. Hervey Barnes, Cleveland; Sec. L. P. Bethel, Kent.

*Pennsylvania State Dental Society*—Will hold its next annual meeting at Eagle's Mere, Pennsylvania, July 9th, 1895. Pres., Dr. J. A. Libbey, Pittsburgh; Cor. Sec., H. E. Roberts, Philadelphia.

*Rhode Island Dental Association*—Pres., C. M. Noble, Providence; Sec., H. W. Gillett, Newport, Meets quarterly, second Tuesday in July, October, January and April. Annual Meeting second Tuesday in July, at Newport.

*South Carolina State Dental Association*—Meets annually. Pres. J. C. Orland, Spartanburg; Cor. Sec. C. B. Colson, Charleston. Next meeting at Spartanburg, October, 1895.

*Tennessee Dental Association*—Meets annually. Next meeting at Knoxville, July 9, 1895. Pres. W. H. Richards, Knoxville; Rec. Sec. W. P. Houston, Lawrenceburg; Cor. Sec. W. J. Morrison, Nashville.

*The Dental Society of the State of New York*—Meets annually on the second Wednesday in May. Next session at Albany, 8th and 9th of May, 1895. Pres. F. T. Van Woert, Brooklyn; Sec. C. S. Butler, Buffalo; Cor. Sec. R. Ottolengui, New York.

*Texas State Dental Association*—Meets in San Antonia, third Tuesday in May, 1894. Pres. A. A. Beville, Waco; Sec. Chas. B. Lewis, Ennis

*Vermont State Dental Society*—Meets annually. Next meeting will be held at Brandon, Vt., third Wednesday in March, 1895. Pres. W. H. Wright, Brandon; Sec. Thos. Mound, Rutland.

*Virginia State Dental Association*—Will meet in 1895. Exact time and place to be decided later. Pres. H. W. Campbell, Suffolk; Sec. J. Hall Moore, Richmond.

*Washington State Dental Society*—Meets Annually. Next meeting will be held at Seattle, in the middle of May, 1895. Pres. B. S. Scott, Ellenburg; Sec., R. B. Gentle, Olympia.

*Wisconsin State Dental Society*—Meets annually. Next meeting will be held at Madison, Wis., the third Tuesday in July, 1895. Pres., Chas. C. Chittenden, Madison; Sec., C. A. Southwell, Milwaukee.

*West Virginia State Society.*—The annual meeting will be held at Parkersburg, on the first Wednesday of October, 1894. Pres. J. N. Mahan, Charleston; Sec. Geo. I. Keener, Morgantown.

### LOCAL SOCIETIES.

*American Academy of Dental Science*—Meets annually in Boston, Mass., on the first Wednesday in October. Pres. J. H. Batchelder, Rec. Sec. William Y. Allen.

*Atktnsonian Dental Society of Chicogo*—Meets second Tuesday each month. Pres. J. G. Reed; Sec. C. E. Meehoff.

*Buffalo Dental Association*—Meets last Monday evening of each month. Annual meeting in June. Pres. F. H. Boswell; Sec. J. A. Stackhouse.

*Brooklyn Dental Society and Second District Society United*—Meets quarterly—Pres. T. H. Holly; Rec. Sec. A. N. Russell, Brooklyn.

*Central Dental Association of Northern New Jersey*—Meets bi-monthly; and annually. The next annual meeting will be held about the middle of February, 1895. Pres. Thomas Moore, Paterson; Sec. Wm. L. Fish, Newark.

*Chicago Dental Society*—Meets monthly. Pres. W. V. B. Ames; Sec. A. H. Peck;

*Chicago Dental Club*—Meets on the fourth Monday of each month. Pres. I. D. Sperling; Sec. E. L. Clifford.

*Cleveland Dental Society.*—Meets the first Monday of each month. Pres. J. R. Bell; Sec. J. F. Stephan.

*Connecticut Valley Dental Society*—Meets annually. Next meeting in Oct. 1895, time and place to be decided later. Pres. C. C. Barker, Meriden, Conn.; Sec. Geo. A. Maxfield, Holyoke, Mass.

*Detroit Dental Society*—Meets monthly, Pres. Geo. L. Field, Sec. A. W. Diack.

*Dental Society of Southwestern Michigan*—Meets at St. Joseph, October 8th and 9th, 1895. Pres. S. M. White; Sec. E. I. Backers.

*Eighth District Dental Society, N. Y.*—Next annual meeting April 24th, 1894, in Buffalo. Pres. F. J. Burkhart, Batavia; Sec., W. V. Grove, Buffalo.

*East Tennessee Dental Association*—Meets annually. Next meeting will be held at Harriman, second Tuesday in June 1895. Pres. F. A. Shotwell, Rogersville; Sec. and Treas. Geo. C. Brause, Harriman.

*Eastern Illinois Dental Society*—Meets annually.

*Fifth District Dental Society of N. Y.*—Meets semi-annually. Next annual meeting will be held in Utica, 2d Tuesday in April, 1895. Pres. Frank Jones, Syracuse; Cor. Sec. A. R. Cooke, Syracuse.

*First District Dental Society of the State of New York*—Meets annually. The next meeting will be held on the second Tuesday of April 1895. Pres., Victor Hugo Jackson, New York City; Sec., Benj. C. Nash, New York City.

*First District Dental Society of Illinois*—Next meeting will be held in Canton, second Tuesday in September, 1895. Pres. W. A. Johnston, Peoria; Sec. W. O. Butter, La Harpe.

*Hayden Dental Society of Chicago*—Meets third Monday each month. Pres. T. E. Powell; Sec. A. J. Oakey.

*Knoxville City Dental Society*—Meets monthly. President, J. T. Cazier; Sec. A. J. Cottrell.

*Minnesota State Dental Society*—Annual Meeting will be held in St. Paul, 2nd Wednesday in Sept., 1895. Pres. C. H. Robinsin, Wabasha; Sec. W. L. Cruttenden, Northfield.

*Mississippi Valley Dental Society*—Meets annually at Cincinnati. Next meeting April, 1897. Pres., Junicus E. Cravens, Indianapolis; Rec. Sec. H. T. Smith, Cincinnati; Cor. Sec. W. S. Lock, Cincinnati, O.

*New England Dental Society*—Meets annually. Next meeting 2nd Thursday in Nov., 1895, in Boston Mass. Pres. J. F. Adams, Worcester, N. H.; Sec. E. O. Kinsman, Cambridge, Mass.

*Northern Illinois Dental Society.*—Meets annually. Next meeting at Aurora, October 17th, 1895. Pres. T. W. Beckwith, Sterling; Sec. J. W. Cormany, Mt. Carroll.

*Northern Indiana Dental Society*—Meets annually third Tuesday in October. Pres. E. J. Church; Sec. C. G Keekn.

*Odontological Society of Pennsylvania*—Meets on the first Saturday evening of each month, at 8 o'clock p. m., at 13th and Arch sts; Pres. Jas. Truman, Rec. Sec. A. W. Deane.

*Odontological Society of New York City*—Meets the third Tuesday of each month. Pres. Dr. A. L. Northrop; Cor. Sec. Dr. George A. Wilson.

*Odontological Society of Cincinnati*—Meets the fourth Friday of each Month; Pres. H. C. Matlack; Sec. W. S. Lock.

*Odontological Society of Chicago*—Meets third Tuesday each month. Pres. S. A. Swasey; Sce. and Treas., Louis Ottofy.

*Odontographic Society of Chicago*—Meets second Monday each month. Pres. Dr. M. Gallie; Sec. H. H. Wilson.

*Pennsylvania Association of Dental Surgeons*—Meets second Tuesday of each month. Pres. H. E. Roberts; Cor. Sec. Theo. F. Chupein.

*Pittsburg Dental Society*—Meets the last Tuesday Evening of each month. Pres. J. B. Williams, Homestead; Sec. N. L. Reinecke.

*Quincy, (Ills.) Dental Society*—Meets Monthly.

*San Francisco Dental Society*—Meets the second Monday evening of each month. Annual Meeting in October. Pres. C. E. Post; Sec. G. N. Van Orden; Cor. Sec. H. G. Richards.

*Sixth District Dental Society of N. Y.*—Meets semi-annually. The annual meeting will be held at Binghamton, the first week in May, 1895. Pres., M. H. Fish, New Berlin; Sec., F. W. McCall, Binghamton.

*Seventh District Dental Society of Ohio*—Meets annually. Next meeting in May, 1894. Pres., O. N. Heise, Cincinnati; Sec., J. R. Callahan, Cincinnati.

*Southern Illinois Dental Society*- Meets annually. Next annual meeting will be held on the 3d Tuesday in October, 1896. Pres., Sec., G. W. Entsminger, Carbondale.

*Southern Minnesota Dental Society*—Next meeting in Mankato, third Tuesday in April, 1895. Pres. S. Bond, Onoka: Sec. F. B. Kremer, Minneapolis.

*Stomatological Club of California*—Meets in Sanfrancisco. Pres. W. J. Younger.

*St. Louis Dental Society*—Meets first and third Tuesday in each month. Pres. J. B. Newby; Cor. Sec. John G Harper; Rec. Sec. F. F. Fletcher.

*The Isaac Knapp Dental Coterie of Fort Wayne, Ind.*—Meets the first Thursday of each month. Pres., W. W. Mungen; Sec., M. A. Mason.

*The Minneapolis Dental Society Monthly*—Pres. E. F. Clark, Sec. E. J. Morrison.

*The Tacoma Dental Society*—Meets monthly. Pres. M.C. Burns; Sec. W.E. Burkhart

*Washington City Dental Society*—Meets 3d Tuesday of each month. Pres. Dr. J. Roland Walton; Sec. Dr. D. Elmer Wiber.

### DIRECTORY OF DENTAL SOCIETIES.

*American Dental Association*—Meets first Tuesday in August, 1896. At Saratoga, N.Y. Pres., J. Y. Crawford, Nashville, Tenn.; Rec. Sec., Geo. H. Cushing, Chicago.

*Southern Dental Association*—Next meeting will be held at Nashville, Tenn., November, 1896. Pres., H. E. Beach, Clarksville, Tenn. Sec., S. W. Foster, Decatur, Ala.

### STATE DENTAL SOCIETIES.

*Alabama Dental Association*—Meets annually. Next meeting at Selma, on the second Tuesday of April, 1896. Pres. A. A. Pearson, Montgomery; Sec. S. W. Foster, Decatur.

*California State Dental Association*—Next meeting will be held in San Francisco, second Tuesday in June, 1896. Pres., L. A. Teague, San Francisco; Rec. Sec., W. Z. King, San Francisco; Cor. Sec., C. E. Post, San Francisco.

*Colorado State Dental Society*—Meets 1st Tuesday in June, 1894, at Glenwood Springs. Pres. P. T. Smith, Denver; Sec., Sarah May Townsend, Denver.

*Connecticut State Dental Society*—Next meeting will be held in Hartford, third Tuesday in May, 1895. Pres. Chas. P. Graham, Middletown; Rec. Sec. Geo. L. Parmele, Hartford.

*Dental Society of the State of Maryland and District of Columbia*—Meets annually. Next meeting at Washington City, on the second Tuesday in October, 1893. Pres. B. F. Coy, of Baltimore: Sec. M. W. Foster, Baltimore.

*Florida State Dental Association*—Meets annually. Next meeting at Tampa, 1st Tuesday in May, 1895. Pres, W. A. McQuaig, Lake City; Cor. Sec. S. W. Allen, Tampa.

*Georgia State Dental Society*—Meets at Indian Spring, in 1895. Exact time to be decided later; Pres. W. W. Hill, Washington; Rec. Sec. S. H. McKee, Americus; Cor. Sec. O. H. McDonald, Atlanta.

*Illinois State Dental Society*—The next meeting will be held at Peoria, Ill., May Second Tuesday, 1897. Pres. C. R. Taylor, Streaton., Sec. Louis Ottofy, Chicago.

*Indiana State Dental Society*—Will meet at Detroit, Mich., on the third Tuesday of June, 1895. Pres. D. S. Harrold, Elwood; Secretary, G. E. Hunt, Indianapolis.

*Iowa State Dental Society*—Meets annually. Next meeting at Iowa City, May, 7th to 10th, inclusive, 1895. Pres. J. S. Kulp, Muscatine; Rec. Sec. F. T. Breene, Iowa City.

*Kentucky State Dental Association*—Meets annually. Next meeting in Louisville, Ky., 3rd Tuesday in June, 1896. Pres., J. H. Letcher, Danville; Sec., J. H. Baldwin, Louisville.

*Kansas State Dental Association*—Pres. C. B. Reed, Topeka; Sec., W. W. West, Topeka. Next meeting will be held at Topeka, second Monday in May, 1895.

*Maine Dental Society*—Will meet on the third Tuesday in July, 1895, in Bangor. Pres. T. E. Tibbetts, Rockland; Sec. F. A. Knowlton, Fairfield.

*Maryland State Dental Association*—Next annual meeting will be held (place not definitely known yet,) in April, 1895. Pres., Chas. C. Harris, Baltimore; Sec, W. W. Dunbracco, Baltimore.

*Massachusetts Dental Society*—Meets annually in Boston, in June. Pres. Josiah W. Ball, Boston; Rec. Sec., E. O. Kinsman, Cambridge.

*Michigan State Dental Association*—Meets annually. Next meeting at Detroit, on the 3rd., Tuesday of June, 1896. Pres., J. Ward House, Grand Rapids; Sec. ————. Treas. Geo. H. Mosher, Jackson.

*Mississippi Dental Association*—Next meeting date to be fixed, 1896, at Jackson, Miss. Pres. T. C. West, Natchez; Sec. L. N. Jeffries, Natchez.

*Minnesota State Society*—Pres. E. B. Weeks, Leitchfield; Sec., H. L. Cruttenden, St. Paul. Will meet in Winona, in September, 1896.

*Missouri Dental Association*—Meets at Bertle Springs, Mo., second Tuesday in July, 1895. Pres., J. T. Fry, Moberly; Sec., W. W. Carter, Sedalia.

*Nebraska State Dental Society.*—Next annual meeting will be held at Norfolk, third Tuesday in May, 1895. Pres., H. W. Shriver, Omaha; Cor. Sec., O. M. Huestis, Nebraska City.

*New Jersey State Dental Society*—Meets annually. The next meeting will be held August, 1896. Pres. R. M. Sanger, E. Orange; Sec. C. A. Meeker, Newark.

*New Hampshire Dental Society*—Meets Annually. Next meeting will be held in Concord, N. H.; time to be decided later. Pres. W. R. Blackstone, Manchester; Sec. Burton C. Russell, Keene.

*North Carolina State Dental Society*—Meets in Salisbury, on the second Tuesday in May, 1895. Pres. H. H. Harper, Kinston; Sec., J. E. Wyche, Greensboro.

*North Dakota State Dental Society*—The next meeting will be held at Fargo, May, 1895. Pres. E. M. Pierce, Hillsboro; Sec. C. L. Rose, Fargo.

*Ohio State Dental Society*—Meets in Columbus, first Tuesday in December, 1896. Pres. Hervey Barnes, Cleveland; Sec. L. P. Bethel, Kent.

*Pennsylvania State Dental Society*—Will hold its next annual meeting at Eagle's Mere, Pennsylvania, July 9th, 1895. Pres., Dr. J. A. Libbey, Pittsburgh; Cor. Sec., H. E. Roberts, Philadelphia.

*Rhode Island Dental Association*—Pres., C. M. Noble, Providence; Sec., H. W. Gillett, Newport, Meets quarterly, second Tuesday in July, October, January and April. Annual Meeting second Tuesday in July, at Newport.

*South Carolina State Dental Association*—Meets annually. Pres. J. C. Orland, Spartanburg; Cor. Sec. C. B. Colson, Charleston. Next meeting at Spartanburg, October, 1895.

*Tennessee Dental Association*—Meets annually. Next meeting at Knoxville, July 9, 1895. Pres. W. H. Richards, Knoxville: Rec Sec. W. P. Houston, Lawrenceburg; Cor. Sec. W. J. Morrison, Nashville.

*The Dental Society of the State of New York*—Meets annually on the second Wednesday in May. Next session at Albany, 8th and 9th of May, 1895. Pres. F. T. Van Woert, Brooklyn; Sec. C. S. Butler, Buffalo; Cor. Sec. R. Ottolengui, New York.

*Texas State Dental Association*—Meets in San Antonia, third Tuesday in May, 1894. Pres. A. A. Beville, Waco; Sec. Chas. B. Lewis, Ennis

*Vermont State Dental Society*—Meets annually. Next meeting will be held at Brandon, Vt., third Wednesday in March, 1895. Pres. W. H. Wright, Brandon; Sec. Thos. Mound, Rutland.

*Virginia State Dental Association*—Will meet in 1895. Exact time and place to be decided later. Pres. H. W. Campbell, Suffolk; Sec. J. Hall Moore, Richmond.

*Washington State Dental Society*—Meets Annually. Next meeting will be held at Seattle, in the middle of May, 1895. Pres. B. S. Scott, Ellenburg; Sec., R. B. Gentle, Olympia.

*Wisconsin State Dental Society*—Meets annually. Next meeting will be held at Madison, Wis., the third Tuesday in July, 1895. Pres., Chas. C. Chittenden, Madison; Sec., C. A. Southwell, Milwaukee.

*West Virginia State Society.*—The annual meeting will be held at Parkersburg, on the first Wednesday of October, 1894. Pres. J. N. Mahan, Charleston; Sec. Geo. I. Keener, Morgantown.

### LOCAL SOCIETIES.

*American Academy of Dental Science*—Meets annually in Boston, Mass., on the first Wednesday in October. Pres. J. H. Batchelder, Rec. Sec. William Y. Allen.

*Atktnsonian Dental Society of Chicogo*—Meets second Tuesday each month. Pres. J. G. Reed; Sec. C. E. Meehoff.

*Buffalo Dental Association*—Meets last Monday evening of each month. Annual meeting in June. Pres. F. H. Boswell; Sec. J. A. Stackhouse.

*Brooklyn Dental Society and Second District Society United*—Meets quarterly—Pres. T. H. Holly; Rec. Sec. A. N. Russell, Brooklyn.

*Central Dental Association of Northern New Jersey*—Meets bi-monthly; and annually. The next annual meeting will be held about the middle of February, 1895. Pres. Thomas Moore, Paterson; Sec. Wm. L. Fish, Newark.

*Chicago Dental Society*—Meets monthly. Pres. W. V. B. Ames; Sec. A. H. Peck;

*Chicago Dental Club*—Meets on the fourth Monday of each month. Pres. I. D. Sperling; Sec. E. L. Clifford.

*Cleveland Dental Society.*—Meets the first Monday of each month. Pres. J. R. Bell; Sec. J. F. Stephan.

*Connecticut Valley Dental Society*—Meets annually. Next meeting in Oct. 1895, time and place to be decided later. Pres. C. C. Barker, Meriden, Conn.; Sec. Geo. A. Maxfield, Holyoke, Mass.

*Detroit Dental Society*—Meets monthly, Pres. Geo. L. Field, Sec. A. W. Diack.

*Dental Society of Southwestern Michigan*—Meets at St. Joseph, October 8th and 9th, 1895. Pres. S. M. White; Sec. E. I. Backers.

*Eighth District Dental Society, N. Y.*—Next annual meeting April 24th, 1894, in Buffalo. Pres. F. J. Burkhart, Batavia; Sec., W. V. Grove, Buffalo.

*East Tennessee Dental Association*—Meets annually. Next meeting will be held at Harriman, second Tuesday in June 1895. Pres. F. A. Shotwell, Rogersville; Sec. and Treas. Geo. C. Brause, Harriman.

*Eastern Illinois Dental Society*—Meets annually.

*Fifth District Dental Society of N. Y.*—Meets semi-annually. Next annual meeting will be held in Utica, 2d Tuesday in April, 1895. Pres. Frank Jones, Syracuse; Cor. Sec. A. R. Cooke, Syracuse.

*First District Dental Society of the State of New York*—Meets annually. The next meeting will be held on the second Tuesday of April 1895. Pres., Victor Hugo Jackson, New York City; Sec., Benj. C. Nash, New York City.

*First District Dental Society of Illinois*—Next meeting will be held in Canton, second Tuesday in September, 1895. Pres. W. A. Johnston, Peoria; Sec. W. O. Butter, La Harpe.

*Hayden Dental Society of Chicago*—Meets third Monday each month. Pres. T. E. Powell; Sec. A. J. Oakey.

*Knoxville City Dental Society*—Meets monthly. President, J. T. Cazier; Sec. A. J. Cottrell.

*Minnesota State Dental Society*—Annual Meeting will be held in St. Paul, 2nd Wednesday in Sept., 1895. Pres. C. H. Robinsin, Wabasha; Sec. W. L. Cruttenden, Northfield.

*Mississippi Valley Dental Society*—Meets annually at Cincinnati. Next meeting April, 1897. Pres., Junicus E. Cravens, Indianapolis; Rec. Sec. H. T. Smith, Cincinnati; Cor. Sec. W. S. Lock, Cincinnati, O.

*New England Dental Society*—Meets annually. Next meeting 2nd Thursday in Nov., 1895, in Boston Mass. Pres. J. F. Adams, Worcester, N. H.; Sec. E. O. Kinsman, Cambridge, Mass.

*Northern Illinois Dental Society.*—Meets annually. Next meeting at Aurora, October 17th, 1895. Pres. T. W. Beckwith, Sterling; Sec. J. W. Cormany, Mt. Carroll.

*Northern Indiana Dental Society*—Meets annually third Tuesday in October. Pres. E. J. Church; Sec. C. G Keekn.

*Odontological Society of Pennsylvania*—Meets on the first Saturday evening of each month, at 8 o'clock p. m., at 13th and Arch sts; Pres. Jas. Truman, Rec. Sec. A. W. Deane.

*Odontological Society of New York City*—Meets the third Tuesday of each month. Pres. Dr. A. L. Northrop; Cor. Sec. Dr. George A. Wilson.

*Odontological Society of Cincinnati*—Meets the fourth Friday of each Month; Pres. H. C. Matlack; Sec. W. S. Lock.

*Odontological Society of Chicago*—Meets third Tuesday each month. Pres. S. A. Swasey; Sce. and Treas., Louis Ottofy.

*Odontographic Society of Chicago*—Meets second Monday each month. Pres. Dr. M. Gallie; Sec. H. H. Wilson.

*Pennsylvania Association of Dental Surgeons*—Meets second Tuesday of each month. Pres. H. E. Roberts; Cor. Sec. Theo. F. Chupein.

*Pittsburg Dental Society*—Meets the last Tuesday Evening of each month. Pres. J. B. Williams, Homestead; Sec. N. L. Reinecke.

*Quincy, (Ills.) Dental Society*—Meets Monthly.

*San Francisco Dental Society*—Meets the second Monday evening of each month. Annual Meeting in October. Pres. C. E. Post; Sec. G. N. Van Orden; Cor. Sec. H. G. Richards.

*Sixth District Dental Society of N. Y.*—Meets semi-annually. The annual meeting will be held at Binghamton, the first week in May, 1895. Pres., M. H. Fish, New Berlin; Sec., F. W. McCall, Binghamton.

*Seventh District Dental Society of Ohio*—Meets annually. Next meeting in May, 1894. Pres., O. N. Heise, Cincinnati; Sec., J. R. Callahan, Cincinnati.

*Southern Illinois Dental Society*—Meets annually. Next annual meeting will be held on the 3d Tuesday in October, 1896. Pres., Sec., G. W. Entsminger, Carbondale.

*Southern Minnesota Dental Society*—Next meeting in Mankato, third Tuesday in April, 1895. Pres. S. Bond, Onoka; Sec. F. B. Kremer, Minneapolis.

*Stomatological Club of California*—Meets in Sanfrancisco. Pres. W. J. Younger.

*St. Louis Dental Society*—Meets first and third Tuesday in each month. Pres. J. B. Newby; Cor. Sec. John G Harper: Rec. Sec. F. F. Fletcher.

*The Isaac Knapp Dental Coterie of Fort Wayne, Ind.*—Meets the first Thursday of each month. Pres., W. W. Mungen; Sec., M. A. Mason.

*The Minneapolis Dental Society Monthly*—Pres. E. F. Clark, Sec. E. J. Morrison.

*The Tacoma Dental Society*—Meets monthly. Pres. M.C. Burns; Sec. W.E. Burkhart

*Washington City Dental Society*—Meets 3d Tuesday of each month. Pres. Dr. J. Roland Walton; Sec. Dr. D. Elmer Wiber.

### DIRECTORY OF DENTAL SOCIETIES.

*American Dental Association*—Meets first Tuesday in August, 1896. At Saratoga, N.Y. Pres., J. Y. Crawford, Nashville, Tenn.; Rec. Sec., Geo. H. Cushing, Chicago.

*Southern Dental Association*—Next meeting will be held at Asheville, N. C., November, 1896. Pres., H. E. Beach, Clarksville, Tenn. Sec., S. W. Foster, Decatur, Ala.

### STATE DENTAL SOCIETIES.

*Alabama Dental Association*—Meets annually. Next meeting at Selma, on the second Tuesday of April, 1896. Pres. A. A. Pearson, Montgomery; Sec. S. W. Foster, Decatur.

*California State Dental Association*—Next meeting will be held in San Francisco, second Tuesday in June, 1896. Pres., L. A. Teague, San Francisco; Rec. Sec., W. Z. King, San Francisco; Cor. Sec., C. E. Post, San Francisco.

*Colorado State Dental Society*—Meets 1st Tuesday in June, 1894, at Glenwood Springs. Pres. P. T. Smith, Denver; Sec., Sarah May Townsend, Denver.

*Connecticut State Dental Society*—Next meeting will be held in Hartford, third Tuesday in May, 1895. Pres. Chas. P. Graham, Middletown; Rec. Sec. Geo. L. Parmele, Hartford.

*Dental Society of the State of Maryland and District of Columbia*—Meets annually. Next meeting at Washington City, on the second Tuesday in October, 1893. Pres. B. F. Coy, of Baltimore: Sec. M. W. Foster, Baltimore.

*Florida State Dental Association*—Meets annually. Next meeting at Tampa, 1st Tuesday in May, 1895. Pres, W. A. McQuaig, Lake City; Cor. Sec. S. W. Allen, Tampa.

*Georgia State Dental Society*—Meets at Indian Spring, in 1895. Exact time to be decided later; Pres. W. W. Hill, Washington; Rec. Sec. S. H. McKee, Americus; Cor. Sec. O. H. McDonald, Atlanta.

*Illinois State Dental Society*—The next meeting will be held at Peoria, Ill., May Second Tuesday, 1897. Pres. C. R. Taylor, Streaton., Sec. Louis Ottofy, Chicago.

*Indiana State Dental Society*—Will meet at Detroit, Mich., on the third Tuesday of June, 1895. Pres. D. S. Harrold, Elwood; Secretary, G. E. Hunt, Indianapolis.

*Iowa State Dental Society*—Meets annually. Next meeting at Iowa City, May, 7th to 10th, inclusive, 1895. Pres. J. S. Kulp, Muscatine; Rec. Sec. F. T. Breene, Iowa City.

*Kentucky State Dental Association*—Meets annually. Next meeting in Louisville, Ky., 3rd Tuesday in June, 1896. Pres., J. H. Letcher, Danville; Sec., J. H. Baldwin, Louisville.

*Kansas State Dental Association*—Pres. C. B. Reed, Topeka; Sec., W. W. West, Topeka. Next meeting will be held at Topeka, second Monday in May, 1895.

*Maine Dental Society*—Will meet on the third Tuesday in July, 1895, in Bangor. Pres. T E. Tibbetts, Rockland; Sec. F. A. Knowlton, Fairfield.

*Maryland State Dental Association*—Next annual meeting will be held (place not definitely known yet,) in April, 1895. Pres., Chas. C. Harris, Baltimore; Sec, W. W. Dunbracco, Baltimore.

*Massachusetts Dental Society*—Meets annually in Boston, in June. Pres. Josiah W. Ball, Boston; Rec. Sec., E. O. Kinsman, Cambridge.

*Michigan State Dental Association*—Meets annually. Next meeting at Detroit, on the 3rd., Tuesday of June, 1896. Pres., J. Ward House, Grand Rapids; Sec. ————. Treas. Geo. H. Mosher, Jackson.

*Mississippi Dental Association*—Next meeting date to be fixed, 1896, at Jackson, Miss. Pres. T. C. West, Natchez; Sec. L. N. Jeffries, Natchez.

*Minnesota State Society*—Pres. E. B. Weeks, Leitchfield; Sec., H. L. Cruttenden, St. Paul. Will meet in Winona, in September, 1896.

*Missouri Dental Association*—Meets at Bertle Springs, Mo., second Tuesday in July, 1895. Pres., J. T. Fry, Moberly; Sec., W. W. Carter, Sedalia.

*Nebraska State Dental Society.*—Next annual meeting will be held at Norfolk, third Tuesday in May, 1895. Pres., H. W. Shriver, Omaha; Cor. Sec., O. M. Huestis, Nebraska City.

*New Jersey State Dental Society*—Meets annually. The next meeting will be held August, 1896. Pres. R. M. Sanger, E. Orange; Sec. C. A. Meeker, Newark.

*New Hampshire Dental Society*—Meets Annually. Next meeting will be held in Concord, N. H.; time to be decided later. Pres. W. R. Blackstone, Manchester; Sec. Burton C. Russell, Keene.

*North Carolina State Dental Society*—Meets in Salisbury, on the second Tuesday in May, 1895. Pres. H. H. Harper, Kinston; Sec., J. E. Wyche, Greensboro.

*North Dakota State Dental Society*—The next meeting will be held at Fargo, May, 1895. Pres. E. M. Pierce, Hillsboro; Sec. C. L. Rose, Fargo.

*Ohio State Dental Society*—Meets in Columbus, first Tuesday in December, 1896. Pres. Hervey Barnes, Cleveland; Sec. L. P. Bethel, Kent.

*Pennsylvania State Dental Society*—Will hold its next annual meeting at Eagle's Mere, Pennsylvania, July 9th, 1895. Pres., Dr. J. A. Libbey, Pittsburgh; Cor. Sec., H. E. Roberts, Philadelphia.

*Rhode Island Dental Association*—Pres., C. M. Noble, Providence; Sec., H. W. Gillett, Newport, Meets quarterly, second Tuesday in July, October, January and April. Annual Meeting second Tuesday in July, at Newport.

*South Carolina State Dental Association*—Meets annually. Pres. J. C. Orland, Spartanburg; Cor. Sec. C. B. Colson, Charleston. Next meeting at Spartanburg, October, 1895.

*Tennessee Dental Association*—Meets annually. Next meeting at Knoxville, July 9, 1895. Pres. W. H. Richards, Knoxville; Rec. Sec. W. P. Houston, Lawrenceburg; Cor. Sec. W. J. Morrison, Nashville.

*The Dental Society of the State of New York*—Meets annually on the second Wednesday in May. Next session at Albany, 8th and 9th of May, 1895. Pres. F. T. Van Woert, Brooklyn; Sec. C. S. Butler, Buffalo; Cor. Sec. R. Ottolengui, New York.

*Texas State Dental Association*—Meets in San Antonia, third Tuesday in May, 1894. Pres. A. A. Beville, Waco; Sec. Chas. B. Lewis, Ennis

*Vermont State Dental Society*—Meets annually. Next meeting will be held at Brandon, Vt., third Wednesday in March, 1895. Pres. W. H. Wright, Brandon; Sec. Thos. Mound, Rutland.

*Virginia State Dental Association*—Will meet in 1895. Exact time and place to be decided later. Pres. H. W. Campbell, Suffolk; Sec. J. Hall Moore, Richmond.

*Washington State Dental Society*—Meets Annually. Next meeting will be held at Seattle, in the middle of May, 1895. Pres. B. S. Scott, Ellenburg; Sec., R. B. Gentle, Olympia.

*Wisconsin State Dental Society*—Meets annually. Next meeting will be held at Madison, Wis., the third Tuesday in July, 1895. Pres., Chas. C. Chittenden, Madison; Sec., C. A. Southwell, Milwaukee.

*West Virginia State Society.*—The annual meeting will be held at Parkersburg, on the first Wednesday of October, 1894. Pres. J. N. Mahan, Charleston; Sec. Geo. I. Keener, Morgantown.

### LOCAL SOCIETIES.

*American Academy of Dental Science*—Meets annually in Boston, Mass., on the first Wednesday in October. Pres. J. H. Batchelder, Rec. Sec. William Y. Allen.

*Buffalo Dental Association*—Meets last Monday evening of each month. Annual meeting in June. Pres. F. H. Boswell; Sec. J. A. Stackhouse.

*Brooklyn Dental Society and Second District Society United*—Meets quarterly—Pres. T. H. Holly; Rec. Sec. A. N. Russell, Brooklyn.

*Central Dental Association of Northern New Jersey*—Meets bi-monthly; and annually. The next annual meeting will be held about the middle of February, 1895. Pres. Thomas Moore, Paterson; Sec. Wm. L. Fish, Newark.

*Chicago Dental Society*—Meets monthly. Pres. Louis Ottofy; Sec. A. H. Peck.

*Chicago Dental Club*—Meets on the fourth Monday of each month. Pres. I. D. Sperling; Sec. E. L. Clifford.

*Cincinnati Acadamy of Dentistry*—Meets monthly. Pres. W. T. McLean; Sec. Wm. Lockman, jr.

*Cleveland Dental Society.*—Meets the first Monday of each month. Pres. J. R. Bell; Sec. J. F. Stephan.

*Connecticut Valley Dental Society*—Meets annually. Next meeting in Oct. 1895, time and place to be decided later. Pres. C. C. Barker, Meriden, Conn.; Sec. Geo. A. Maxfield, Holyoke, Mass.

*Detroit Dental Society*—Meets monthly, Pres. Geo. L. Field, Sec. A. W. Diack.

*Dental Society of Southwestern Michigan*—Meets at St. Joseph, October 8th and 9th, 1895. Pres. S. M. White; Sec. E. I. Backers.

*Eighth District Dental Society, N. Y.*—Next annual meeting April 24th, 1894, in Buffalo. Pres. F. J. Burkhart, Batavia; Sec., W. V. Grove, Buffalo

*East Tennessee Dental Association*—Meets annually. Next meeting will be held at Harriman, second Tuesday in June 1895. Pres. F. A. Shotwell, Rogersville; Sec. and Treas. Geo. C. Brause, Harriman.

*Eastern Illinois Dental Society*—Meets annually.

*Fifth District Dental Society of N. Y.*—Meets semi-annually. Next annual meeting will be held in Utica, 2d Tuesday in April, 1895. Pres. Frank Jones, Syracuse; Cor. Sec. A. R. Cooke, Syracuse.

*First District Dental Society of the State of New York*—Meets annually. The next meeting will be held on the second Tuesday of April 1895. Pres., Victor Hugo Jackson, New York City; Sec., Benj. C. Nash, New York City.

*First District Dental Society of Illinois*—Next meeting will be held in Streetor, second Tuesday in September, 1895. Pres. L. W. Skidmore, Moline; Sec. W. O. Butter, La Harpe.

*Hayden Dental Society of Chicago*—Meets third Monday each month. Pres. J. J. Comelins; Sec. A. J. Oakey.

*Knoxville City Dental Society*—Meets monthly. President, J. T. Cazier; Sec. A. J. Cottrell.

*Minnesota State Dental Society*—Annual Meeting will be held in St. Paul, 2nd Wednesday in Sept., 1895. Pres. C. H. Robinsin, Wabasha; Sec. W. L. Cruttenden, Northfield.

*Mississippi Valley Dental Society*—Meets annually at Cincinnati. Next meeting April, 1897. Pres., Junicus E. Cravens, Indianapolis; Rec. Sec. H. T. Smith, Cincinnati; Cor. Sec. W. S. Lock, Cincinnati, O.

*New England Dental Society*—Meets annually. Next meeting 2nd Thursday in Nov., 1895, in Boston Mass. Pres. J. F. Adams, Worcester, N. H.; Sec. E. O. Kinsman, Cambridge, Mass.

*Northern Illinois Dental Society*—Meets annually. Next meeting at Aurora, October 17th, 1895. Pres. T. W. Beckwith, Sterling; Sec. J. W. Cormany, Mt. Carroll.

*Northern Indiana Dental Society*—Meets annually third Tuesday in October. Pres. E. J. Church; Sec. C. G. Keekn.

*Odontological Society of Pennsylvania*—Meets on the first Saturday evening of each month, at 8 o'clock p. m., at 13th and Arch sts; Pres. Jas. Truman, Rec. Sec. A. W. Deane.

*Odontological Society of New York City*—Meets the third Tuesday of each month. Pres. Dr. A. L. Northrop; Cor. Sec. Dr. George A. Wilson.

*Odontological Society of Cincinnati*—Meets the fourth Friday of each Month; Pres. H. C. Matlack; Sec. W. S. Lock.

*Odontological Society of Chicago*—Meets third Tuesday each month. Pres. C. S. Case; Sec and Treas., L. L. Davis

*Odontographic Society of Chicago*—Meets second Monday each month. Pres. Dr. M. Gallie; Sec. H. H. Wilson.

*Pennsylvania Association of Dental Surgeons*—Meets second Tuesday of each month. Pres. H. E. Roberts; Cor. Sec. Theo. F. Chupein.

*Pittsburg Dental Society*—Meets the last Tuesday Evening of each month. Pres. J. B. Williams, Homestead; Sec. N. L. Reinecke.

*Quincy, (Ills.) Dental Society*—Meets Monthly.

*San Francisco Dental Society*—Meets the second Monday evening of each month. Annual Meeting in October. Pres. C. E. Post; Sec. G. N. Van Orden; Cor. Sec. H. G. Richards.

*Sixth District Dental Society of N. Y.*—Meets semi-annually. The annual meeting will be held at Binghamton, the first week in May, 1895. Pres., M. H. Fish, New Berlin; Sec., F. W. McCall, Binghamton.

*Seventh District Dental Society of Ohio*—Meets annually. Next meeting in May, 1894. Pres., O. N. Heise, Cincinnati; Sec., J. R. Callahan, Cincinnati.

*Southern Illinois Dental Society*—Meets annually. Next annual meeting will be held on the 3d Tuesday in October, 1896. Pres., Sec., G. W. Entsminger, Carbondale.

*Southern Minnesota Dental Society*—Next meeting in Mankato, third Tuesday in April, 1895. Pres. S. Bond, Onoka: Sec. F. B. Kremer, Minneapolis.

*Stomatological Club of California*—Meets in Sanfrancisco. Pres. W. J. Younger.

*St. Louis Dental Society*—Meets first and third Tuesday in each month. Pres. J. B. Newby; Cor. Sec. John G Harper: Rec. Sec. F. F. Fletcher.

*The Isaac Knapp Dental Coterie of Fort Wayne, Ind.*—Meets the first Thursday of each month. Pres., W. W. Mungen; Sec., M. A. Mason.

*The Minneapolis Dental Society Monthly*—Pres. E. F. Clark, Sec. E. J. Morrison.

*The Tacoma Dental Society*—Meets monthly. Pres. M.C. Burns; Sec. W. E. Burkhart.

*Washington City Dental Society*—Meets 3d Tuesday of each month. Pres. Dr. J. Roland Walton; Sec. Dr. D. Elmer Wiber.

### DIRECTORY OF DENTAL SOCIETIES.

*American Dental Association*—Meets first Tuesday in August, 1897. At Old Point Comfort, Va. Pres., James Trnman, Philadelphia, Pa.; Rec. Sec., Geo. H. Cushing, Chicago.

*Southern Dental Association*—Next meeting will be held at Old Point Comfort, Va. First Tuesday in August 1898. Pres., H. E. Beach, Clarksville, Tenn. Sec., S. W. Foster, Decatur, Ala

### STATE DENTAL SOCIETIES.

*Alabama Dental Association*—Meets annually. Next meeting at Selma, on the second Tuesday of April, 1896. Pres. A. A. Pearson, Montgomery; Sec. S. W. Foster, Decatur.

*California State Dental Association*—Next meeting will be held in San Francisco, second Tuesday in June, 1896. Pres., L. A. Teague, San Francisco; Rec. Sec., W. Z. King, San Francisco; Cor. Sec., C. E. Post, San Francisco.

*Colorado State Dental Society*—Meets 1st Tuesday in June, 1894, at Glenwood Springs. Pres. P. T. Smith, Denver; Sec., Sarah May Townsend, Denver.

*Connecticut State Dental Society*—Next meeting will be held in Hartford, third Tuesday in May, 1895. Pres. Chas. P. Graham, Middletown; Rec. Sec. Geo. L. Parmele, Hartford.

*Dental Society of the State of Maryland and District of Columbia*—Meets annually. Next meeting at Washington City, on the second Tuesday in October, 1893. Pres. B. F. Coy, of Baltimore: Sec. M. W. Foster, Baltimore.

*Florida State Dental Association*—Meets annually. Next meeting at Tampa, 1st Tuesday in May, 1895. Pres, W. A. McQuaig, Lake City; Cor. Sec. S. W. Allen, Tampa.

*Georgia State Dental Society*—Meets at Indian Spring, in 1895. Exact time to be decided later; Pres. W. W. Hill, Washington; Rec. Sec. S. H. McKee, Americus; Cor. Sec. O. H. McDonald, Atlanta.

*Illinois State Dental Society*—The next meeting will be held at Peoria, Ill., May Second Tuesday, 1897. Pres. C. R. Taylor, Streaton., Sec. Louis Ottofy, Chicago.

*Indiana State Dental Society*—Will meet at Detroit, Mich., on the third Tuesday of June, 1895. Pres. D. S. Harrold, Elwood; Secretary, G. E. Hunt, Indianapolis.

*Iowa State Dental Society*—Meets annually. Next meeting at Iowa City, May, 7th to 10th, inclusive, 1895. Pres. J. S. Kulp, Muscatine; Rec. Sec. F. T. Breene, Iowa City.

*Kentucky State Dental Association*—Meets annually. Next meeting in Louisville, Ky., 3rd Tuesday in June, 1896. Pres., J. H. Letcher, Danville; Sec., J. H. Baldwin, Louisville.

*Kansas State Dental Association*—Pres. C. B. Reed, Topeka; Sec., W. W. West, Topeka. Next meeting will be held at Topeka, second Monday in May, 1895.

*Maine Dental Society*—Will meet on the third Tuesday in July, 1895, in Bangor. Pres. T E. Tibbetts, Rockland; Sec. F. A. Knowlton, Fairfield.

*Maryland State Dental Association*—Next annual meeting will be held (place not definitely known yet,) in April, 1895. Pres., Chas. C. Harris, Baltimore; Sec, W. W. Dunbracco, Baltimore.

*Massachusetts Dental Society*—Meets annually in Boston, in June. Pres. Josiah W. Ball, Boston; Rec. Sec., E. O. Kinsman, Cambridge.

*Michigan State Dental Association*—Meets annually. Next meeting at Detroit, on the 3rd., Tuesday of June, 1896. Pres., J. Ward House, Grand Rapids; Sec. ———. Treas. Geo. H. Mosher, Jackson.

*Mississippi Dental Association*—Next meeting date to be fixed, 1896, at Jackson, Miss. Pres. T. C. West, Natchez; Sec. L. N. Jeffries, Natchez.

*Minnesota State Society*—Pres. E. B. Weeks, Leitchfield; Sec., H. L. Cruttenden, St. Paul. Will meet in Winona, in September, 1896.

*Missouri Dental Association*—Meets at Bertle Springs, Mo., second Tuesday in July, 1895. Pres., J. T. Fry, Moberly; Sec., W. W. Carter, Sedalia.

*Nebraska State Dental Society*.—Next annual meeting will be held at Norfolk, third Tuesday in May, 1895. Pres., H. W. Shriver, Omaha; Cor. Sec., O. M. Huestis, Nebraska City.

*New Jersey State Dental Society*—Meets annually. The next meeting will be held August, 1897. Pres. Harvey Irdell, New Brunswick; Sec. C. A. Meeker, Newark.

*New Hampshire Dental Society*—Meets Annually. Next meeting will be held in Concord, N. H.; time to be decided later. Pres. W. R. Blackstone, Manchester; Sec. Burton C. Russell, Keene.

*North Carolina State Dental Society*—Meets in Salisbury, on the second Tuesday in May, 1895. Pres. H. H. Harper, Kinston; Sec., J. E. Wyche, Greensboro.

*North Dakota State Dental Society*—The next meeting will be held at Fargo, May, 1895. Pres. E. M. Pierce, Hillsboro; Sec. C. L. Rose, Fargo.

*Ohio State Dental Society*—Meets in Columbus, first Tuesday in December, 1896. Pres. Henry Barnes, Cleveland; Sec. L. P. Bethel, Kent.

*Pennsylvania State Dental Society*—Will hold its next annual meeting at Eagle's Mere, Pennsylvania, July 9th, 1895. Pres., Dr. J. A. Libbey, Pittsburgh; Cor. Sec., H. E. Roberts, Philadelphia.

*Rhode Island Dental Association*—Pres., C. M. Noble, Providence; Sec., H. W. Gillett, Newport, Meets quarterly, second Tuesday in July, October, January and April. Annual Meeting second Tuesday in July, at Newport.

*South Carolina State Dental Association*—Meets annually. Pres. J. C. Orland, Spartanburg; Cor. Sec. C. B. Colson, Charleston. Next meeting at Spartanburg, October, 1895.

*Tennessee Dental Association*—Meets annually. Next meeting at Knoxville, July 9, 1895. Pres. W. H. Richards, Knoxville: Rec. Sec. W. P. Houston, Lawrenceburg; Cor. Sec. W. J. Morrison, Nashville.

*The Dental Society of the State of New York*—Meets annually on the second Wednesday in May. Next session at Albany, 8th and 9th of May, 1897. Pres. F. T. Van Woert, Brooklyn; Sec. C. S. Butler, Buffalo; Cor. Sec. R. Ottolengui, New York.

*Texas State Dental Association*—Meets in San Antonia, third Tuesday in May, 1894. Pres. A. A. Beville, Waco; Sec. Chas. B. Lewis, Ennis

*Vermont State Dental Society*—Meets annually. Next meeting will be held at Brandon, Vt., third Wednesday in March, 1895. Pres. W. H. Wright, Brandon; Sec. Thos. Mound, Rutland.

*Virginia State Dental Association*—Will meet in 1895. Exact time and place to be decided later. Pres. H. W. Campbell, Suffolk; Sec. J. Hall Moore, Richmond.

*Washington State Dental Society*—Meets Annually. Next meeting will be held at Seattle, in the middle of May, 1895. Pres. B. S. Scott, Ellenburg; Sec., R. B. Gentle, Olympia.

*Wisconsin State Dental Society*—Meets annually. Next meeting will be held at Madison, Wis., the third Tuesday in July, 1895. Pres., Chas. C. Chittenden, Madison; Sec., C. A. Southwell, Milwaukee.

*West Virginia State Society.*—The annual meeting will be held at Parkersburg, on the first Wednesday of October, 1894. Pres. J. N. Mahan, Charleston; Sec. Geo. I. Keener, Morgantown.

### LOCAL SOCIETIES.

*American Academy of Dental Science*—Meets annually in Boston, Mass., on the first Wednesday in October. Pres. J. H. Batchelder, Rec. Sec. William Y. Allen.

*Buffalo Dental Association*—Meets last Monday evening of each month. Annual meeting in June. Pres. F. H. Boswell; Sec. J. A. Stackhouse.

*Brooklyn Dental Society and Second District Society United*—Meets quarterly—Pres. T. H. Holly; Rec. Sec. A. N. Russell, Brooklyn.

*Central Dental Association of Northern New Jersey*—Meets bi-monthly; and annually. The next annual meeting will be held about the middle of February, 1895. Pres. Thomas Moore, Paterson; Sec. Wm. L. Fish, Newark.

*Chicago Dental Society*—Meets monthly. Pres. Louis Ottofy; Sec. A. H. Peck.

*Chicago Dental Club*—Meets on the fourth Monday of each month. Pres. I. D. Sperling; Sec. E. L. Clifford.

*Cincinnati Acadamy of Dentistry*—Meets monthly. Pres. W. T. McLean; Sec. Wm. Lockman, jr.

*Cleveland Dental Society.*—Meets the first Monday of each month. Pres. J. R. Bell; Sec. J. F. Stephan.

*Connecticut Valley Dental Society*—Meets annually. Next meeting in Oct. 1895, time and place to be decided later. Pres. C. C. Barker, Meriden, Conn.; Sec. Geo. A. Maxfield, Holyoke, Mass.

*Detroit Dental Society*—Meets monthly, Pres. Geo. L. Field, Sec. A. W. Diack.

*Dental Society of Southwestern Michigan*—Meets at St. Joseph, October 8th and 9th, 1895. Pres. S. M. White; Sec. E. I. Backers.

*Eighth District Dental Society, N. Y.*—Next annual meeting April 24th, 1894, in Buffalo. Pres. F. J. Burkhart, Batavia; Sec., W. V. Grove, Buffalo

*East Tennessee Dental Association*—Meets annually. Next meeting will be held at Harriman, second Tuesday in June 1895. Pres. F. A. Shotwell, Rogersville; Sec. and Treas. Geo. C. Brause, Harriman.

*Eastern Illinois Dental Society*—Meets annually.

*Fifth District Dental Society of N. Y.*—Meets semi-annually. Next annual meeting will be held in Utica, 2d Tuesday in April, 1895. Pres. Frank Jones, Syracuse; Cor. Sec. A. R. Cooke, Syracuse.

*First District Dental Society of the State of New York*—Meets annually. The next meeting will be held on the second Tuesday of April 1895. Pres., Victor Hugo Jackson, New York City; Sec., Benj. C. Nash, New York City.

*First District Dental Society of Illinois*—Next meeting will be held in Streetor, second Tuesday in September, 1895. Pres. L. W. Skidmore, Moline; Sec. W. O. Butter, La Harpe.

*Hayden Dental Society of Chicago*—Meets third Monday each month. Pres. J. J. Comelins; Sec. A. J. Oakey.

*Knoxville City Dental Society*—Meets monthly. President, J. T. Cazier; Sec. A. J. Cottrell.

*Minnesota State Dental Society*—Annual Meeting will be held in St. Paul, 2nd Wednesday in Sept., 1895. Pres. C. H. Robinsin, Wabasha; Sec. W. L. Cruttenden, Northfield.

*Mississippi Valley Dental Society*—Meets annually at Cincinnati. Next meeting April, 1897. Pres., Junicus E. Cravens, Indianapolis; Rec. Sec. H. T. Smith, Cincinnati; Cor. Sec. W. S. Lock, Cincinnati, O.

*New England Dental Society*—Meets annually. Next meeting 2nd Thursday in Nov., 1895, in Boston Mass. Pres. J. F. Adams, Worcester, N. H.; Sec. E. O. Kinsman, Cambridge, Mass.

*Northern Illinois Dental Society*—Meets annually. Next meeting at Aurora, October 17th, 1895. Pres. T. W. Beckwith, Sterling; Sec. J. W. Cormany, Mt. Carroll.

*Northern Indiana Dental Society*—Meets annually third Tuesday in October. Pres. E. J. Church; Sec. C. G. Keekn.

*Odontological Society of Pennsylvania*—Meets on the first Saturday evening of each month, at 8 o'clock p. m., at 13th and Arch sts; Pres. Jas. Truman, Rec. Sec. A W. Deane.

*Odontological Society of New York City*—Meets the third Tuesday of each month. Pres. Dr. A. L. Northrop; Cor. Sec. Dr. George A. Wilson.

*Odontological Society of Cincinnati*—Meets the fourth Friday of each Month; Pres. H. C. Matlack; Sec. W. S. Lock.

*Odontological Society of Chicago*—Meets third Tuesday each month. Pres. C. S. Case; Sec and Treas., L. L. Davis

*Odontographic Society of Chicago*—Meets second Monday each month. Pres. Dr. M. Gallie; Sec. H. H. Wilson.

*Pennsylvania Association of Dental Surgeons*—Meets second Tuesday of each month. Pres. H. E. Roberts; Cor. Sec. Theo. F. Chupein.

*Pittsburg Dental Society*—Meets the last Tuesday Evening of each month. Pres. J. B. Williams, Homestead; Sec. N. L. Reinecke.

*Quincy, (Ills.) Dental Society*—Meets Monthly.

*San Francisco Dental Society*—Meets the second Monday evening of each month. Annual Meeting in October. Pres. C. E. Post; Sec. G. N. Van Orden; Cor. Sec. H. G. Richards.

*Sixth District Dental Society of N. Y.*—Meets semi-annually. The annual meeting will be held at Binghamton, the first week in May, 1895. Pres., M. H. Fish, New Berlin; Sec., F. W. McCall, Binghamton.

*Seventh District Dental Society of Ohio*—Meets annually. Next meeting in May, 1894. Pres., O. N. Heise, Cincinnati; Sec., J. R. Callahan, Cincinnati.

*Southern Illinois Dental Society*—Meets annually. Next annual meeting will be held on the 3d Tuesday in October, 1896. Pres., Sec., G. W. Entsminger, Carbondale.

*Southern Minnesota Dental Society*—Next meeting in Mankato, third Tuesday in April, 1895. Pres. S. Bond, Onoka; Sec. F. B. Kremer, Minneapolis.

*Stomatological Club of California*—Meets in Sanfrancisco. Pres. W. J. Younger.

*St. Louis Dental Society*—Meets first and third Tuesday in each month. Pres. J. B. Newby; Cor. Sec. John G Harper; Rec. Sec. F. F. Fletcher.

*The Isaac Knapp Dental Coterie of Fort Wayne, Ind.*—Meets the first Thursday of each month. Pres., W. W. Mungen; Sec., M. A. Mason.

*The Minneapolis Dental Society Monthly*—Pres. E. F. Clark, Sec. E. J. Morrison.

*The Tacoma Dental Society*—Meets monthly. Pres. M.C. Burns; Sec. W. E. Burkhart

*Washington City Dental Society*—Meets 3d Tuesday of each month. Pres. Dr. J. Roland Walton; Sec. Dr. D. Elmer Wiber.

### DIRECTORY OF DENTAL SOCIETIES.

*American Dental Association*—Meets first Tuesday in August, 1897. At Old Point Comfort, Va. Pres., James Trnman, Philadelphia, Pa.; Rec. Sec., Geo. H. Cushing, Chicago.

*Southern Dental Association*—Next meeting will be held at Old Point Comfort, Va. First Tuesday in August 1898. Pres., W. H. Richards, Knoxville, Tenn. Sec., C. L. Alexander, Charlotte, N. C.

### STATE DENTAL SOCIETIES.

*Alabama Dental Association*—Meets annually. Next meeting at Selma, on the second Tuesday of April, 1896. Pres. A. A. Pearson, Montgomery; Sec. S. W. Foster, Decatur.

*California State Dental Association*—Next meeting will be held in San Francisco, second Tuesday in June, 1896. Pres., L. A. Teague, San Francisco; Rec. Sec., W. Z. King, San Francisco; Cor. Sec., C. E. Post, San Francisco.

*Colorado State Dental Society*—Meets 1st Tuesday in June, 1894, at Glenwood Springs. Pres. P. T. Smith, Denver; Sec., Sarah May Townsend, Denver.

*Connecticut State Dental Society*—Next meeting will be held in Hartford, third Tuesday in May, 1895. Pres. Chas. P. Graham, Middletown; Rec. Sec. Geo. L. Parmele, Hartford.

*Dental Society of the State of Maryland and District of Columbia*—Meets annually. Next meeting at Washington City, on the second Tuesday in October, 1893. Pres. B. F. Coy, of Baltimore; Sec. M. W. Foster, Baltimore.

*Florida State Dental Association*—Meets annually. Next meeting at Tampa, 1st Tuesday in May, 1895. Pres, W. A. McQuaig, Lake City; Cor. Sec. S. W. Allen, Tampa.

*Georgia State Dental Society*—Meets at Indian Spring, in 1895. Exact time to be decided later; Pres. W. W Hill, Washington; Rec. Sec. S. H. McKee, Americus; Cor. Sec. O. H. McDonald, Atlanta.

*Illinois State Dental Society*—The next meeting will be held at Peoria, Ill., May Second Tuesday, 1897. Pres. C. R. Taylor, Streaton., Sec. Louis Ottofy, Chicago.

*Indiana State Dental Society*—Will meet at Detroit, Mich., on the third Tuesday of June, 1895. Pres. D. S. Harrold, Elwood; Secretary, G. E. Hunt, Indianapolis.

*Iowa State Dental Society*—Meets annually. Next meeting at Iowa City, May, 7th to 10th, inclusive, 1895. Pres. J. S. Kulp, Muscatine; Rec. Sec. F. T. Breene, Iowa City.

*Kentucky State Dental Association*—Meets annually. Next meeting in Louisville, Ky., 3rd Tuesday in June, 1896. Pres., J. H. Letcher, Danville; Sec., J. H. Baldwin, Louisville.

*Kansas State Dental Association*—Pres. C. B. Reed, Topeka; Sec., W. W. West, Topeka. Next meeting will be held at Topeka, second Monday in May, 1895.

*Maine Dental Society*—Will meet on the third Tuesday in July, 1895, in Bangor. Pres. T E. Tibbetts, Rockland; Sec. F. A. Knowlton, Fairfield.

*Maryland State Dental Association*—Next annual meeting will be held (place not definitely known yet,) in April, 1895. Pres., Chas. C. Harris, Baltimore; Sec, W. W. Dunbracco, Baltimore.

*Massachusetts Dental Society*—Meets annually in Boston, in June. Pres. Josiah W. Ball, Boston; Rec. Sec., E. O. Kinsman, Cambridge.

*Michigan State Dental Association*—Meets annually. Next meeting at Detroit, on the 3rd., Tuesday of June, 1896. Pres., J. Ward House, Grand Rapids; Sec. ———. Treas. Geo. H. Mosher, Jackson.

*Mississippi Dental Association*—Next meeting date to be fixed, 1896, at Jackson, Miss. Pres. T. C. West, Natchez; Sec. L. N. Jeffries, Natchez.

*Minnesota State Society*—Pres. E. B. Weeks, Leitchfield; Sec., H. L. Cruttenden, St. Paul. Will meet in Winona, in September, 1896.

*Missouri Dental Association*—Meets at Bertle Springs, Mo., second Tuesday in July, 1895. Pres., J. T. Fry, Moberly; Sec., W. W. Carter, Sedalia.

*Nebraska State Dental Society.*—Next annual meeting will be held at Norfolk, third Tuesday in May, 1895. Pres., H. W. Shriver, Omaha; Cor. Sec., O. M. Huestis, Nebraska City.

*New Jersey State Dental Society*—Meets annually. The next meeting will be held August, 1897. Pres. Harvey Irdell, New Brunswick; Sec. C. A. Meeker, Newark.

*New Hampshire Dental Society*—Meets Annually. Next meeting will be held in Concord, N. H.; time to be decided later. Pres. W. R. Blackstone, Manchester; Sec. Burton C. Russell, Keene.

*North Carolina State Dental Society*—Meets in Salisbury, on the second Tuesday in May, 1895. Pres. H. H. Harper, Kinston; Sec., J. E. Wyche, Greensboro.

*North Dakota State Dental Society*—The next meeting will be held at Fargo, May, 1895. Pres. E. M. Pierce, Hillsboro; Sec. C. L. Rose, Fargo.

*Ohio State Dental Society*—Meets in Columbus, first Tuesday in December, 1896. Pres. Henry Barnes, Cleveland; Sec. L. P. Bethel, Kent.

*Pennsylvania State Dental Society*—Will hold its next annual meeting at Eagle's Mere, Pennsylvania, July 9th, 1895. Pres., Dr. J. A. Libbey, Pittsburgh; Cor. Sec., H. E. Roberts, Philadelphia.

*Rhode Island Dental Association*—Pres., C. M. Noble, Providence; Sec., H. W. Gillett, Newport, Meets quarterly, second Tuesday in July, October, January and April. Annual Meeting second Tuesday in July, at Newport.

*South Carolina State Dental Association*—Meets annually. Pres. J. C. Orland, Spartanburg; Cor. Sec. C. B. Colson, Charleston. Next meeting at Spartanburg, October, 1895.

*Tennessee Dental Association*—Meets annually. Next meeting at Knoxville, July 9, 1895. Pres. W. H. Richards, Knoxville; Rec Sec. W. P. Houston, Lawrenceburg; Cor. Sec. W. J. Morrison, Nashville.

*The Dental Society of the State of New York*—Meets annually on the second Wednesday in May. Next session at Albany, 8th and 9th of May, 1897. Pres. F. T. Van Woert, Brooklyn; Sec. C. S. Butler, Buffalo; Cor. Sec. R. Ottolengui, New York.

*Texas State Dental Association*—Meets in San Antonia, third Tuesday in May, 1894. Pres. A. A. Beville, Waco; Sec. Chas. B. Lewis, Ennis

*Vermont State Dental Society*—Meets annually. Next meeting will be held at Brandon, Vt., third Wednesday in March, 1895. Pres. W. H. Wright, Brandon; Sec. Thos. Mound, Rutland.

*Virginia State Dental Association*—Will meet in 1895. Exact time and place to be decided later. Pres. H. W. Campbell, Suffolk; Sec. J. Hall Moore, Richmond.

*Washington State Dental Society*—Meets Annually. Next meeting will be held at Seattle, in the middle of May, 1895. Pres. B. S. Scott, Ellenburg; Sec., R. B. Gentle, Olympia.

*Wisconsin State Dental Society*—Meets annually. Next meeting will be held at Madison, Wis., the third Tuesday in July, 1895. Pres., Chas. C. Chittenden, Madison; Sec., C. A. Southwell, Milwaukee.

*West Virginia State Society.*—The annual meeting will be held at Charleston, on the third Wednesday of January, 1897. Pres. Chas H. Bartlett, Parkersburg; Sec, G. W. Storer, Wheeling.

### LOCAL SOCIETIES.

*American Academy of Dental Science*—Meets annually in Boston, Mass., on the first Wednesday in October. Pres. J. H. Batchelder, Rec. Sec. William Y. Allen.

*Buffalo Dental Association*—Meets last Monday evening of each month. Annual meeting in June. Pres. F. H. Boswell; Sec. J. A. Stackhouse.

*Brooklyn Dental Society and Second District Society United*—Meets quarterly—Pres. T. H. Holly; Rec. Sec. A. N. Russell, Brooklyn.

*Central Dental Association of Northern New Jersey*—Meets bi-monthly; and annually. The next annual meeting will be held about the middle of February, 1895. Pres. Thomas Moore, Paterson; Sec. Wm. L. Fish, Newark.

*Chicago Dental Society*—Meets monthly. Pres. Louis Ottofy; Sec. A. H. Peck.

*Chicago Dental Club*—Meets on the fourth Monday of each month. Pres. I. D. Sperling; Sec. E. L. Clifford.

*Cincinnati Acadamy of Dentistry*—Meets monthly. Pres. W. T. McLean; Sec. Wm. Lockman, jr.

*Cleveland Dental Society.*—Meets the first Monday of each month. Pres. J. R. Bell; Sec. J. F. Stephan.

*Connecticut Valley Dental Society*—Meets annually. Next meeting in Oct. 1895, time and place to be decided later. Pres. C. C. Barker, Meriden, Conn.; Sec. Geo. A. Maxfield, Holyoke, Mass.

*Detroit Dental Society*—Meets monthly, Pres. Geo. L. Field, Sec. A. W. Diack.

*Dental Society of Southwestern Michigan*—Meets at St. Joseph, October 8th and 9th, 1895. Pres. S. M. White; Sec. E. I. Backers.

*Eighth District Dental Society, N. Y.*—Next annual meeting April 24th, 1894, in Buffalo. Pres. F. J. Burkhart, Batavia; Sec., W. V. Grove, Buffalo

*East Tennessee Dental Association*—Meets annually. Next meeting will be held at Harriman, second Tuesday in June 1895. Pres. F. A. Shotwell, Rogersville; Sec. and Treas. Geo. C. Brause, Harriman.

*Eastern Illinois Dental Society*—Meets annually.

*Fifth District Dental Society of N. Y.*—Meets semi-annually. Next annual meeting will be held in Utica, 2d Tuesday in April, 1895. Pres. Frank Jones, Syracuse; Cor. Sec. A. R. Cooke, Syracuse.

*First District Dental Society of the State of New York*—Meets annually. The next meeting will be held on the second Tuesday of April 1895. Pres., Victor Hugo Jackson, New York City; Sec., Benj. C. Nash, New York City.

*First District Dental Society of Illinois*—Next meeting will be held in Streetor, second Tuesday in September, 1895. Pres. L. W. Skidmore, Moline; Sec. W. O. Butter, La Harpe.

*Hayden Dental Society of Chicago*—Meets third Monday each month. Pres. J. J. Comelins; Sec. A. J. Oakey.

*Knoxville City Dental Society*—Meets monthly. President, J. T. Cazier; Sec. A. J. Cottrell.

*Minnesota State Dental Society*—Annual Meeting will be held in St. Paul, 2nd Wednesday in Sept., 1895. Pres. C. H. Robinsin, Wabasha; Sec. W. L. Cruttenden, Northfield.

*Mississippi Valley Dental Society*—Meets annually at Cincinnati. Next meeting April, 1897. Pres., Junicus E. Cravens, Indianapolis; Rec. Sec. H. T. Smith, Cincinnati; Cor. Sec. W. S. Lock, Cincinnati, O.

*New England Dental Society*—Meets annually. Next meeting 2nd Thursday in Nov., 1895, in Boston Mass. Pres. J. F. Adams, Worcester, N. H.; Sec. E. O. Kinsman, Cambridge, Mass.

*Northern Illinois Dental Society*—Meets annually. Next meeting at Aurora, October 17th, 1895. Pres. T. W. Beckwith, Sterling; Sec. J. W. Cormany, Mt. Carroll.

*Northern Indiana Dental Society*—Meets annually third Tuesday in October. Pres. E. J. Church; Sec. C. G. Keekn.

*Odontological Society of Pennsylvania*—Meets on the first Saturday evening of each month, at 8 o'clock p. m., at 13th and Arch sts; Pres. Jas. Truman, Rec. Sec. A W. Deane.

*Odontological Society of New York City*—Meets the third Tuesday of each month. Pres. Dr. A. L. Northrop; Cor. Sec. Dr. George A. Wilson.

*Odontological Society of Cincinnati*—Meets the fourth Friday of each Month; Pres. H. C. Matlack; Sec. W. S. Lock.

*Odontological Society of Chicago*—Meets third Tuesday each month. Pres. C. S. Case; Sec and Treas., L. L. Davis

*Odontographic Society of Chicago*—Meets second Monday each month. Pres. Dr. M. Gallie; Sec. H. H. Wilson.

*Pennsylvania Association of Dental Surgeons*—Meets second Tuesday of each month. Pres. H. E. Roberts; Cor. Sec. Theo. F. Chupein.

*Pittsburg Dental Society*—Meets the last Tuesday Evening of each month. Pres. J. B. Williams, Homestead; Sec. N. L. Reinecke.

*Quincy, (Ills.) Dental Society*—Meets Monthly.

*San Francisco Dental Society*—Meets the second Monday evening of each month. Annual Meeting in October. Pres. C. E. Post; Sec. G. N. Van Orden; Cor. Sec. H. G. Richards.

*Sixth District Dental Society of N. Y.*—Meets semi-annually. The annual meeting will be held at Binghamton, the first week in May, 1895. Pres., M. H. Fish, New Berlin; Sec., F. W. McCall, Binghamton.

*Seventh District Dental Society of Ohio*—Meets annually. Next meeting in May, 1894. Pres., O. N. Heise, Cincinnati; Sec., J. R. Callahan, Cincinnati.

*Southern Illinois Dental Society* Meets annually. Next annual meeting will be held on the 3d Tuesday in October, 1896. Pres., Sec., G. W. Entsminger, Carbondale.

*Southern Minnesota Dental Society*—Next meeting in Mankato, third Tuesday in April, 1895. Pres. S. Bond, Onoka: Sec. F. B. Kremer, Minneapolis.

*Stomatological Club of California*—Meets in Sanfrancisco. Pres. W. J. Younger.

*St. Louis Dental Society*—Meets first and third Tuesday in each month. Pres. J. B. Newby; Cor. Sec. John G Harper; Rec. Sec. F. F. Fletcher.

*The Isaac Knapp Dental Coterie of Fort Wayne, Ind.*—Meets the first Thursday of each month. Pres., W. W. Mungen; Sec., M. A. Mason.

*The Minneapolis Dental Society Monthly*—Pres. E. F. Clark, Sec. E. J. Morrison.

*The Tacoma Dental Society*—Meets monthly. Pres. M.C. Burns; Sec. W. E. Burkhart

*Washington City Dental Society*—Meets 3d Tuesday of each month. Pres. Dr. J. Roland Walton; Sec. Dr. D. Elmer Wiber.

### DIRECTORY OF DENTAL SOCIETIES.

*American Dental Association*—Meets first Tuesday in August, 1897. At Old Point Comfort, Va. Pres., James Trnman, Philadelphia, Pa.; Rec. Sec., Geo. H. Cushing, Chicago.

*Southern Dental Association*—Next meeting will be held at Old Point Comfort, Va. First Tuesday in August 1898. Pres., W. H. Richards, Knoxville, Tenn. Sec., C. L. Alexander, Charlotte, N. C.

### STATE DENTAL SOCIETIES.

*Alabama Dental Association*—Meets annually. Next meeting at Selma, on the second Tuesday of April, 1896. Pres. A. A. Pearson, Montgomery; Sec. S. W. Foster, Decatur.

*California State Dental Association*—Next meeting will be held in San Francisco, second Tuesday in June, 1896. Pres., L. A. Teague, San Francisco; Rec. Sec., W. Z. King, San Francisco; Cor. Sec., C. E. Post, San Francisco.

*Colorado State Dental Society*—Meets 1st Tuesday in June, 1894, at Glenwood Springs. Pres. P. T. Smith, Denver; Sec., Sarah May Townsend, Denver.

*Connecticut State Dental Society*—Next meeting will be held in Hartford, third Tuesday in May, 1895. Pres. Chas. P. Graham, Middletown; Rec. Sec. Geo. L. Parmele, Hartford.

*Dental Society of the State of Maryland and District of Columbia*—Meets annually. Next meeting at Washington City, on the second Tuesday in October, 1893. Pres. B. F. Coy. of Baltimore: Sec. M. W. Foster, Baltimore.

*Florida State Dental Association*—Meets annually. Next meeting at Tampa, 1st Tuesday in May, 1895. Pres, W. A. McQuaig, Lake City; Cor. Sec. S. W. Allen, Tampa.

*Georgia State Dental Society*—Meets at Indian Spring, in 1895. Exact time to be decided later; Pres. W. W. Hill, Washington; Rec. Sec. S. H. McKee, Americus; Cor. Sec. O. H. McDonald, Atlanta.

*Illinois State Dental Society*—The next meeting will be held at Peoria, Ill., May Second Tuesday, 1897. Pres. C. R. Taylor, Streaton., Sec. Louis Ottofy, Chicago.

*Indiana State Dental Society*—Will meet at Detroit, Mich., on the third Tuesday of June, 1895. Pres. D. S. Harrold, Elwood; Secretary, G. E. Hunt, Indianapolis.

*Iowa State Dental Society*—Meets annually. Next meeting at Iowa City, May, 7th to 10th, inclusive, 1895. Pres. J. S. Kulp, Muscatine; Rec. Sec. F. T. Breene, Iowa City.

*Kentucky State Dental Association*—Meets annually. Next meeting in Louisville, Ky., 3rd Tuesday in June, 1896. Pres., J. H. Letcher, Danville; Sec., J. H. Baldwin, Louisville.

*Kansas State Dental Association*—Pres. C. B. Reed, Topeka; Sec., W. W. West, Topeka. Next meeting will be held at Topeka, second Monday in May, 1895.

*Maine Dental Society*—Will meet on the third Tuesday in July, 1895, in Bangor. Pres. T E. Tibbetts, Rockland; Sec. F. A. Knowlton, Fairfield.

*Maryland State Dental Association*—Next annual meeting will be held (place not definitely known yet,) in April, 1895. Pres., Chas. C. Harris, Baltimore; Sec, W. W. Dunbracco, Baltimore.

*Massachusetts Dental Society*—Meets annually in Boston, in June. Pres. Josiah W. Ball, Boston; Rec. Sec., E. O. Kinsman, Cambridge.

*Michigan State Dental Association*—Meets annually. Next meeting at Detroit, on the 3rd., Tuesday of June, 1896. Pres., J. Ward House, Grand Rapids; Sec. ————. Treas. Geo. H. Mosher, Jackson.

*Mississippi Dental Association*—Next meeting date to be fixed, 1895, at Jackson, Miss. Pres. T. C. West, Natchez; Sec. L. N. Jeffries, Natchez.

*Minnesota State Society*—Pres. E. B. Weeks, Leitchfield; Sec., H. L. Cruttenden, St. Paul. Will meet in Winona, in September, 1896.

*Missouri Dental Association*—Meets at Bertle Springs, Mo., second Tuesday in July, 1895. Pres., J. T. Fry, Moberly; Sec., W. W. Carter, Sedalia.

*Nebraska State Dental Society.*—Next annual meeting will be held at Norfolk, third Tuesday in May, 1895. Pres., H. W. Shriver, Omaha; Cor. Sec., O. M. Huestis, Nebraska City.

*New Jersey State Dental Society*—Meets annually. The next meeting will be held August, 1897. Pres. Harvey Irdell, New Brunswick; Sec. C. A. Meeker, Newark.

*New Hampshire Dental Society*—Meets Annually. Next meeting will be held in Concord, N. H.; time to be decided later. Pres. W. R. Blackstone, Manchester; Sec. Burton C. Russell, Keene.

*North Carolina State Dental Society*—Meets in Salisbury, on the second Tuesday in May, 1895. Pres. H. H. Harper, Kinston; Sec., J. E. Wyche, Greensboro.

*North Dakota State Dental Society*—The next meeting will be held at Fargo, May, 1895. Pres. E. M. Pierce, Hillsboro; Sec. C. L. Rose, Fargo.

*Ohio State Dental Society*—Meets in Columbus, first Tuesday in December, 1896. Pres. Henry Barnes, Cleveland; Sec. L. P. Bethel, Kent.

*Pennsylvania State Dental Society*—Will hold its next annual meeting at Eagle's Mere, Pennsylvania, July 9th, 1895. Pres., Dr. J. A. Libbey, Pittsburgh; Cor. Sec., H. E. Roberts, Philadelphia.

*Rhode Island Dental Association*—Pres., C. M. Noble, Providence; Sec., H. W. Gillett, Newport, Meets quarterly, second Tuesday in July, October, January and April. Annual Meeting second Tuesday in July, at Newport.

*South Carolina State Dental Association*—Meets annually. Pres. J. C. Orland, Spartanburg; Cor. Sec. C. B. Colson, Charleston. Next meeting at Spartanburg, October, 1895.

*Tennessee Dental Association*—Meets annually. Next meeting at Knoxville, July 9, 1895. Pres. W. H. Richards, Knoxville: Rec Sec. W. P. Houston, Lawrenceburg; Cor. Sec. W. J. Morrison, Nashville.

*The Dental Society of the State of New York*—Meets annually on the second Wednesday in May. Next session at Albany, 8th and 9th of May, 1897. Pres. F. T. Van Woert, Brooklyn; Sec. C. S. Butler, Buffalo; Cor. Sec. R. Ottolengui, New York.

*Texas State Dental Association*—Meets in San Antonia, third Tuesday in May, 1894. Pres. A. A. Beville, Waco; Sec. Chas. B. Lewis, Ennis

*Vermont State Dental Society*—Meets annually. Next meeting will be held at Brandon, Vt., third Wednesday in March, 1895. Pres. W. H. Wright, Brandon; Sec. Thos. Mound, Rutland.

*Virginia State Dental Association*—Will meet in 1895. Exact time and place to be decided later. Pres. H. W. Campbell, Suffolk; Sec. J. Hall Moore, Richmond.

*Washington State Dental Society*—Meets Annually. Next meeting will be held at Seattle, in the middle of May, 1895. Pres. B. S. Scott, Ellenburg; Sec., R. B. Gentle, Olympia.

*Wisconsin State Dental Society*—Meets annually. Next meeting will be held at Madison, Wis., the third Tuesday in July, 1895. Pres., Chas. C. Chittenden, Madison; Sec., C. A. Southwell, Milwaukee.

*West Virginia State Society.*—The annual meeting will be held at Charleston. on the third Wednesday of January, 1897. Pres. Chas. H. Bartlett, Parkersburg; Sec., G. W. Storer, Wheeling.

### LOCAL SOCIETIES.

*American Academy of Dental Science*—Meets annually in Boston, Mass., on the first Wednesday in October. Pres. J. H. Batchelder, Rec. Sec. William Y. Allen.

*Buffalo Dental Association*—Meets last Monday evening of each month. Annual meeting in June. Pres. F. H. Boswell; Sec. J. A. Stackhouse.

*Brooklyn Dental Society and Second District Society United*—Meets quarterly—Pres. T. H. Holly; Rec. Sec. A. N. Russell, Brooklyn.

*Central Dental Association of Northern New Jersey*—Meets bi-monthly; and annually. The next annual meeting will be held about the middle of February, 1895. Pres. Thomas Moore, Paterson; Sec. Wm. L. Fish, Newark.

*Chicago Dental Society*—Meets monthly. Pres. Louis Ottofy; Sec. A. H. Peck.

*Chicago Dental Club*—Meets on the fourth Monday of each month. Pres. I. D. Sperling; Sec. E. L. Clifford.

*Cincinnati Acadamy of Dentistry*—Meets monthly. Pres. W. T. McLean; Sec. Wm. Lockman, jr.

*Cleveland Dental Society.*—Meets the first Monday of each month. Pres. J. R. Bell; Sec. J. F. Stephan.

*Connecticut Valley Dental Society*—Meets annually. Next meeting in Oct. 1895, time and place to be decided later. Pres. C. C. Barker, Meriden, Conn.; Sec. Geo. A. Maxfield, Holyoke, Mass.

*Detroit Dental Society*—Meets monthly, Pres. Geo. L. Field, Sec. A. W. Diack.

*Dental Society of Southwestern Michigan*—Meets at St. Joseph, October 8th and 9th, 1895. Pres. S. M. White; Sec. E. I. Backers.

*Eighth District Dental Society, N. Y.*—Next annual meeting April 24th, 1894, in Buffalo. Pres. F. J. Burkhart, Batavia; Sec., W. V. Grove, Buffalo

*East Tennessee Dental Association*—Meets annually. Next meeting will be held at Harriman, second Tuesday in June 1895. Pres. F. A. Shotwell, Rogersville; Sec. and Treas. Geo. C. Brause, Harriman.

*Eastern Illinois Dental Society*—Meets annually.

*Fifth District Dental Society of N. Y.*—Meets semi-annually. Next annual meeting will be held in Utica, 2d Tuesday in April, 1895. Pres. Frank Jones, Syracuse; Cor. Sec. A. R. Cooke, Syracuse.

*First District Dental Society of the State of New York*—Meets annually. The next meeting will be held on the second Tuesday of April 1895. Pres., Victor Hugo Jackson, New York City; Sec., Benj. C. Nash, New York City.

*First District Dental Society of Illinois*—Next meeting will be held in Streetor, second Tuesday in September, 1895. Pres. L. W. Skidmore, Moline; Sec. W. O. Butter, La Harpe.

*Hayden Dental Society of Chicago*—Meets third Monday each month. Pres. J. J. Comelins; Sec. A. J. Oakey.

*Knoxville City Dental Society*—Meets monthly. President, J. T. Cazier; Sec. A. J. Cottrell.

*Minnesota State Dental Society*—Annual Meeting will be held in St. Paul, 2nd Wednesday in Sept., 1895. Pres. C. H. Robinsin, Wabasha; Sec. W. L. Cruttenden, Northfield.

*Mississippi Valley Dental Society*—Meets annually at Cincinnati. Next meeting April, 1897. Pres., Junicus E. Cravens, Indianapolis; Rec. Sec. H. T. Smith, Cincinnati; Cor. Sec. W. S. Lock, Cincinnati, O.

*New England Dental Society*—Meets annually. Next meeting 2nd Thursday in Nov., 1895, in Boston Mass. Pres. J. F. Adams, Worcester, N. H.; Sec. E. O. Kinsman, Cambridge, Mass.

*Northern Illinois Dental Society*—Meets annually. Next meeting at Aurora, October 17th, 1895. Pres. T. W. Beckwith, Sterling; Sec. J. W. Cormany, Mt. Carroll.

*Northern Indiana Dental Society*—Meets annually third Tuesday in October. Pres. E. J. Church; Sec. C. G. Keekn.

*Odontological Society of Pennsylvania*—Meets on the first Saturday evening of each month, at 8 o'clock p. m., at 13th and Arch sts; Pres. Jas. Truman, Rec. Sec. A. W. Deane.

*Odontological Society of New York City*—Meets the third Tuesday of each month. Pres. Dr. A. L. Northrop; Cor. Sec. Dr. George A. Wilson.

*Odontological Society of Cincinnati*—Meets the fourth Friday of each Month; Pres. H. C. Matlack; Sec. W. S. Lock.

*Odontological Society of Chicago*—Meets third Tuesday each month. Pres. C. S. Case; Sec and Treas., L. L. Davis

*Odontographic Society of Chicago*—Meets second Monday each month. Pres. Dr. M. Gallie; Sec. H. H. Wilson.

*Pennsylvania Association of Dental Surgeons*—Meets second Tuesday of each month. Pres. H. E. Roberts; Cor. Sec. Theo. F. Chupein.

*Pittsburg Dental Society*—Meets the last Tuesday Evening of each month. Pres. J. B. Williams, Homestead; Sec. N. L. Reinecke.

*Quincy, (Ills.) Dental Society*—Meets Monthly.

*San Francisco Dental Society*—Meets the second Monday evening of each month. Annual Meeting in October. Pres. C. E. Post; Sec. G. N. Van Orden; Cor. Sec. H. G. Richards.

*Sixth District Dental Society of N. Y.*—Meets semi-annually. The annual meeting will be held at Binghamton, the first week in May, 1895. Pres., M. H. Fish, New Berlin; Sec., F. W. McCall, Binghamton.

*Seventh District Dental Society of Ohio*—Meets annually. Next meeting in May, 1894. Pres., O. N. Heise, Cincinnati; Sec., J. R. Callahan, Cincinnati.

*Southern Illinois Dental Society*—Meets annually. Next annual meeting will be held on the 3d Tuesday in October, 1896. Pres., Sec., G. W. Entsminger, Carbondale.

*Southern Minnesota Dental Society*—Next meeting in Mankato, third Tuesday in April, 1895. Pres. S. Bond, Onoka; Sec. F. B. Kremer, Minneapolis.

*Stomatological Club of California*—Meets in Sanfrancisco. Pres. W. J. Younger.

*St. Louis Dental Society*—Meets first and third Tuesday in each month. Pres. J. B. Newby; Cor. Sec. John G Harper; Rec. Sec. F. F. Fletcher.

*The Isaac Knapp Dental Coterie of Fort Wayne, Ind.*—Meets the first Thursday of each month. Pres., W. W. Mungen; Sec., M. A. Mason.

*The Minneapolis Dental Society Monthly*—Pres. E. F. Clark, Sec. E. J. Morrison.

*The Tacoma Dental Society*—Meets monthly. Pres. M.C. Burns; Sec. W.E. Burkhart.

*Washington City Dental Society*—Meets 3d Tuesday of each month. Pres. Dr. J Roland Walton; Sec. Dr. D. Elmer Wiber.

### DIRECTORY OF DENTAL SOCIETIES.

*American Dental Association*—Meets first Tuesday in August, 1897. At Old Point Comfort, Va. Pres., James Trnman, Philadelphia, Pa.; Rec. Sec. Geo. H. Cushing, Chicago.

*Southern Dental Association*—Next meeting will be held at Old Point Comfort, Va. First Tuesday in August 1898. Pres., W. H. Richards, Knoxville, Tenn. Sec., C. L. Alexander, Charlotte, N. C.

### STATE DENTAL SOCIETIES.

*Alabama Dental Association*—Meets annually. Next meeting at Selma, on the second Tuesday of April, 1896. Pres. A. A. Pearson, Montgomery; Sec. S. W. Foster, Decatur.

*California State Dental Association*—Next meeting will be held in San Francisco, second Tuesday in June, 1896. Pres., L. A. Teague, San Francisco; Rec. Sec., W. Z. King, San Francisco; Cor. Sec., C. E. Post, San Francisco.

*Colorado State Dental Society*—Meets 1st Tuesday in June, 1894, at Glenwood Springs. Pres. P. T. Smith, Denver; Sec., Sarah May Townsend, Denver.

*Connecticut State Dental Society*—Next meeting will be held in Hartford, third Tuesday in May, 1895. Pres. Chas. P. Graham, Middletown; Rec. Sec. Geo. L. Parmele, Hartford.

*Dental Society of the State of Maryland and District of Columbia*—Meets annually. Next meeting at Washington City, on the second Tuesday in October, 1893. Pres. B. F. Coy, of Baltimore: Sec. M. W. Foster, Baltimore.

*Florida State Dental Association*—Meets annually. Next meeting at Tampa, 1st Tuesday in May, 1895. Pres, W. A. McQuaig, Lake City; Cor. Sec. S. W. Allen, Tampa.

*Georgia State Dental Society*—Meets at Indian Spring, in 1895. Exact time to be decided later; Pres. W. W. Hill, Washington; Rec. Sec. S. H. McKee, Americus; Cor. Sec. O. H. McDonald, Atlanta.

*Illinois State Dental Society*—The next meeting will be held at Peoria, Ill., May Second Tuesday, 1897. Pres. C. R. Taylor, Streaton., Sec. Louis Ottofy, Chicago.

*Indiana State Dental Society*—Will meet at Detroit, Mich., on the third Tuesday of June, 1895. Pres. D. S. Harrold, Elwood; Secretary, G. E. Hunt, Indianapolis.

*Iowa State Dental Society*—Meets annually. Next meeting at Iowa City, May, 7th to 10th, inclusive, 1895. Pres. J. S. Kulp, Muscatine; Rec. Sec. F. T. Breene, Iowa City.

*Kentucky State Dental Association*—Meets annually. Next meeting in Louisville, Ky., 3rd Tuesday in June, 1896. Pres., J. H. Letcher, Danville; Sec., J. H. Baldwin, Louisville.

*Kansas State Dental Association*—Pres. C. B. Reed, Topeka; Sec., W. W. West, Topeka. Next meeting will be held at Topeka, second Monday in May, 1895.

*Maine Dental Society*—Will meet on the third Tuesday in July, 1895, in Bangor. Pres. T E. Tibbetts, Rockland; Sec. F. A. Knowlton, Fairfield.

*Maryland State Dental Association*—Next annual meeting will be held (place not definitely known yet,) in April, 1895. Pres., Chas. C. Harris, Baltimore; Sec, W. W. Dunbracco, Baltimore.

*Massachusetts Dental Society*—Meets annually in Boston, in June. Pres. Josiah W. Ball, Boston; Rec. Sec., E. O. Kinsman, Cambridge.

*Michigan State Dental Association*—Meets annually. Next meeting at Detroit, on the 3rd., Tuesday of June, 1896. Pres., J. Ward House, Grand Rapids; Sec. ———. Treas. Geo. H. Mosher, Jackson.

*Mississippi Dental Association*—Next meeting date to be fixed, 1896, at Jackson, Miss. Pres. T. C. West, Natchez; Sec. L. N. Jeffries, Natchez.

*Minnesota State Society*—Pres. E. B. Weeks, Leitchfield; Sec., H. L. Cruttenden, St. Paul. Will meet in Winona, in September, 1896.

*Missouri Dental Association*—Meets at Bertle Springs, Mo., second Tuesday in July, 1895. Pres., J. T. Fry, Moberly; Sec., W. W. Carter, Sedalia.

*Nebraska State Dental Society.*—Next annual meeting will be held at Norfolk, third Tuesday in May, 1895. Pres., H. W. Shriver, Omaha; Cor. Sec., O. M. Huestis, Nebraska City.

*New Jersey State Dental Society*—Meets annually. The next meeting will be held August, 1897. Pres. Harvey Irdell, New Brunswick; Sec. C. A. Meeker, Newark.

*New Hampshire Dental Society*—Meets Annually. Next meeting will be held in Concord, N. H.; time to be decided later. Pres. W. R. Blackstone, Manchester; Sec. Burton C. Russell, Keene.

*North Carolina State Dental Society*—Meets in Salisbury, on the second Tuesday in May, 1895. Pres. H. H. Harper, Kinston; Sec., J. E. Wyche, Greensboro.

*North Dakota State Dental Society*—The next meeting will be held at Fargo, May, 1895. Pres. E. M. Pierce, Hillsboro; Sec. C. L. Rose, Fargo.

*Ohio State Dental Society*—Meets in Columbus, first Tuesday in December, 1896. Pres. Henry Barnes, Cleveland; Sec. L. P. Bethel, Kent.

*Pennsylvania State Dental Society*—Will hold its next annual meeting at Eagle's Mere, Pennsylvania, July 9th, 1895. Pres., Dr. J. A. Libbey, Pittsburgh; Cor. Sec., H. E. Roberts, Philadelphia.

*Rhode Island Dental Association*—Pres., C. M. Noble, Providence; Sec., H. W. Gillett, Newport, Meets quarterly, second Tuesday in July, October, January and April. Annual Meeting second Tuesday in July, at Newport.

*South Carolina State Dental Association*—Meets annually. Pres. J. C. Orland, Spartanburg; Cor. Sec. C. B. Colson, Charleston. Next meeting at Spartanburg, October, 1895.

*Tennessee Dental Association*—Meets annually. Next meeting at Knoxville, July 9, 1895. Pres. W. H. Richards, Knoxville: Rec. Sec. W. P. Houston, Lawrenceburg; Cor. Sec. W. J. Morrison, Nashville.

*The Dental Society of the State of New York*—Meets annually on the second Wednesday in May. Next session at Albany, 8th and 9th of May, 1897. Pres. F. T. Van Woert, Brooklyn; Sec. C. S. Butler, Buffalo; Cor. Sec. R. Ottolengui, New York.

*Texas State Dental Association*—Meets in San Antonia, third Tuesday in May, 1894. Pres. A. A. Beville, Waco; Sec. Chas. B. Lewis, Ennis

*Vermont State Dental Society*—Meets annually. Next meeting will be held at Brandon, Vt., third Wednesday in March, 1895. Pres. W. H. Wright, Brandon; Sec. Thos. Mound, Rutland.

*Virginia State Dental Association*—Will meet in 1895. Exact time and place to be decided later. Pres. H. W. Campbell, Suffolk; Sec. J. Hall Moore, Richmond.

*Washington State Dental Society*—Meets Annually. Next meeting will be held at Seattle, in the middle of May, 1895. Pres. B. S. Scott, Ellenburg; Sec., R. B. Gentle, Olympia.

*Wisconsin State Dental Society*—Meets annually. Next meeting will be held at Madison, Wis., the third Tuesday in July, 1895. Pres., Chas. C. Chittenden, Madison; Sec., C. A. Southwell, Milwaukee.

*West Virginia State Society.*—The annual meeting will be held at Charleston, on the third Wednesday of January, 1897. Pres. Chas. H. Bartlett, Parkersburg; Sec., G. W. Storer, Wheeling.

### LOCAL SOCIETIES.

*American Academy of Dental Science*—Meets annually in Boston, Mass., on the first Wednesday in October. Pres. J. H. Batchelder, Rec. Sec. William Y. Allen.

*Buffalo Dental Association*—Meets last Monday evening of each month. Annual meeting in June. Pres. F. H. Boswell; Sec. J. A. Stackhouse.

*Brooklyn Dental Society and Second District Society United*—Meets quarterly—Pres. T. H. Holly; Rec. Sec. A. N. Russell, Brooklyn.

*Central Dental Association of Northern New Jersey*—Meets bi-monthly; and annually. The next annual meeting will be held about the middle of February, 1895. Pres. Thomas Moore, Paterson; Sec. Wm. L. Fish, Newark.

*Chicago Dental Society*—Meets monthly. Pres. Louis Ottofy; Sec. A. H. Peck.

*Chicago Dental Club*—Meets on the fourth Monday of each month. Pres. I. D. Sperling; Sec. E. L. Clifford.

*Cincinnati Acadamy of Dentistry*—Meets monthly. Pres. W. T. McLean; Sec. Wm. Lockman, jr.

*Cleveland Dental Society.*—Meets the first Monday of each month. Pres. J. R. Bell; Sec. J. F. Stephan.

*Connecticut Valley Dental Society*—Meets annually. Next meeting in Oct. 1895, time and place to be decided later. Pres. C. C. Barker, Meriden, Conn.; Sec. Geo. A. Maxfield, Holyoke, Mass.

*Detroit Dental Society*—Meets monthly, Pres. Geo. L. Field, Sec. A. W. Diack.

*Dental Society of Southwestern Michigan*—Meets at St. Joseph, October 8th and 9th, 1895. Pres. S. M. White; Sec. E. I. Backers.

*Eighth District Dental Society, N. Y.*—Next annual meeting April 24th, 1894, in Buffalo. Pres. F. J. Burkhart, Batavia; Sec., W. V. Grove, Buffalo

*East Tennessee Dental Association*—Meets annually. Next meeting will be held at Harriman, second Tuesday in June 1895. Pres. F. A. Shotwell, Rogersville; Sec. and Treas. Geo. C. Brause, Harriman.

*Eastern Illinois Dental Society* - Meets annually.

*Fifth District Dental Society of N. Y.*—Meets semi-annually. Next annual meeting will be held in Utica, 2d Tuesday in April, 1895. Pres. Frank Jones, Syracuse; Cor. Sec. A. R. Cooke, Syracuse.

*First District Dental Society of the State of New York*—Meets annually. The next meeting will be held on the second Tuesday of April 1895. Pres., Victor Hugo Jackson, New York City; Sec., Benj. C. Nash, New York City.

*First District Dental Society of Illinois*—Next meeting will be held in Streetor, second Tuesday in September, 1895. Pres. L. W. Skidmore, Moline; Sec. W. O. Butter, La Harpe.

*Hayden Dental Society of Chicago*—Meets third Monday each month. Pres. J. J. Comelins; Sec. A. J. Oakey.

*Knoxville City Dental Society*—Meets monthly. President, J. T. Cazier; Sec. A. J. Cottrell.

*Minnesota State Dental Society*—Annual Meeting will be held in St. Paul, 2nd Wednesday in Sept., 1895. Pres. C. H. Robinsin, Wabasha; Sec. W. L. Cruttenden, Northfield.

*Mississippi Valley Dental Society*—Meets annually at Cincinnati. Next meeting April, 1897. Pres., Junicus E. Cravens, Indianapolis; Rec. Sec. H. T. Smith, Cincinnati; Cor. Sec. W. S. Lock, Cincinnati, O.

*New England Dental Society*—Meets annually. Next meeting 2nd Thursday in Nov., 1895, in Boston Mass. Pres. J. F. Adams, Worcester, N. H.; Sec. E. O. Kinsman, Cambridge, Mass.

*Northern Illinois Dental Society*.—Meets annually. Next meeting at Aurora, October 17th, 1895. Pres. T. W. Beckwith, Sterling; Sec. J. W. Cormany, Mt. Carroll.

*Northern Indiana Dental Society*—Meets annually third Tuesday in October. Pres. E. J. Church; Sec. C. G. Keekn.

*Odontological Society of Pennsylvania*—Meets on the first Saturday evening of each month, at 8 o'clock p. m., at 13th and Arch sts; Pres. Jas. Truman, Rec. Sec. A W. Deane.

*Odontological Society of New York City*—Meets the third Tuesday of each month. Pres. Dr. A. L. Northrop; Cor. Sec. Dr. George A. Wilson.

*Odontological Society of Cincinnati*—Meets the fourth Friday of each Month; Pres. H. C. Matlack; Sec. W. S. Lock.

*Odontological Society of Chicago*—Meets third Tuesday each month. Pres. C. S. Case; Sec and Treas., L. L. Davis

*Odontographic Society of Chicago*—Meets second Monday each month. Pres. Dr. M. Gallie; Sec. H. H. Wilson.

*Pennsylvania Association of Dental Surgeons*—Meets second Tuesday of each month. Pres. H. E. Roberts; Cor. Sec. Theo. F. Chupein.

*Pittsburg Dental Society*—Meets the last Tuesday Evening of each month. Pres. J. B. Williams, Homestead; Sec. N. L. Reinecke.

*Quincy, (Ills.) Dental Society*—Meets Monthly.

*San Francisco Dental Society*—Meets the second Monday evening of each month. Annual Meeting in October. Pres. C. E. Post; Sec. G. N. Van Orden; Cor. Sec. H. G. Richards.

*Sixth District Dental Society of N. Y.*—Meets semi-annually. The annual meeting will be held at Binghamton, the first week in May, 1895. Pres., M. H. Fish, New Berlin; Sec., F. W. McCall, Binghamton.

*Seventh District Dental Society of Ohio*—Meets annually. Next meeting in May, 1894. Pres., O. N. Heise, Cincinnati; Sec., J. R. Callahan, Cincinnati.

*Southern Illinois Dental Society* - Meets annually. Next annual meeting will be held on the 3d Tuesday in October, 1896. Pres., Sec., G. W. Entsminger, Carbondale.

*Southern Minnesota Dental Society*—Next meeting in Mankato, third Tuesday in April, 1895. Pres. S. Bond, Onoka; Sec. F. B. Kremer, Minneapolis.

*Stomatological Club of California*—Meets in Sanfrancisco. Pres. W. J. Younger.

*St. Louis Dental Society*—Meets first and third Tuesday in each month. Pres. J. B. Newby; Cor. Sec. John G Harper; Rec. Sec. F. F. Fletcher.

*The Isaac Knapp Dental Coterie of Fort Wayne, Ind.*—Meets the first Thursday of each month. Pres., W. W. Mungen; Sec., M. A. Mason.

*The Minneapolis Dental Society Monthly*—Pres. E. F. Clark, Sec. E. J. Morrison.

*The Tacoma Dental Society*—Meets monthly. Pres. M.C. Burns; Sec. W.E. Burkhart

*Washington City Dental Society*—Meets 3d Tuesday of each month. Pres. Dr. J Roland Walton; Sec. Dr. D. Elmer Wiber.

### CONTENTS.

### THE DENTAL REGISTER,

A MONTHLY MAGAZINE DEVOTED TO THE INTERESTS OF THE

**DENTAL PROFESSION.**

*Terms Reduced to* $2.00 *Per Year. Single Copies,* 25 *Cents.*

**EDITED BY PROF. J. TAFT, D.D.S.**

—AND—

**Published by SAM'L A. CROCKER & CO., Ohio Dental and Surgical Depot.**

The volume commences in January and closes in December.

The REGISTER being issued monthly is always fresh, therefore Indispensable to the practitioner who desires to keep posted up with the new inventions and improvements which are daily developed. Neither expense nor effort will be spared to give value and variety to its contents. Articles will be illustrated with well executed engravings whenever they will add to the interest or assist to explanation.

Its extensive circulation and frequency of issue make it one of the BEST DENTAL ADVERTISING MEDIUMS in the United States.

All subscriptions must begin with January or July.

Local or special notices, five lines or less, will be inserted for $3 each insertion; over five lines, 50 cts. per line.

All advertising bills payable in advance, or at furthest, after the issue of the first number containing the advertisement. All transient advertisements to be paid for in advance.

☞CONTRIBUTIONS SOLICITED.

Exclusively Editorial Communications should be addressed to the Editor.

The latest number of the REGISTER may be ordered with or without other good[illegible] any time, and all letters should be addressed to [illegible]

